# Beckmann Rearrangement of Ketoxime Catalyzed by *N*-methyl-imidazolium Hydrosulfate

**DOI:** 10.3390/molecules23071764

**Published:** 2018-07-18

**Authors:** Hongyu Hu, Xuting Cai, Zhuying Xu, Xiaoyang Yan, Shengxian Zhao

**Affiliations:** Xingzhi College, Zhejiang Normal University, Jinhua 321004, China; huhongyu22@126.com (H.H.); CxuT1998@163.com (X.C.); hu841124@126.com (Z.X.)

**Keywords:** Beckmann rearrangement, ketoxime, acidic ionic liquid, catalysis

## Abstract

Beckmann rearrangement of ketoxime catalyzed by acidic ionic liquid-*N*-methyl-imidazolium hydrosulfate was studied. Rearrangement of benzophenone oxime gave the desirable product with 45% yield at 90 °C. When co-catalyst P_2_O_5_ was added, the yield could be improved to 91%. The catalyst could be reused three cycles with the same efficiency. Finally, reactions of other ketoximes were also investigated.

## 1. Introduction

Over the past years, amide derivatives have received much attention owing to their broad range of applications in many fields such as the pharmaceutical industry, chemical biology, the agrochemical industry, engineering plastics, and so on [1,2,3,4,5,6]. Various approaches have been developed for the synthesis of amide compounds including nucleophilic acyl substitution reactions with amines [7], Staudinger ligation [8], Schmidt reaction [9] and Beckmann rearrangement [10]. However, generations of large amounts of undesired by-products and corrosive phenomenon associated with common acid (H_2_SO_4_ and SOCl_2_) based on liquid phase protocols provide a challenging task for chemists to develop alternative methods [11,12]. A variety of alternative routes [13,14,15,16] based on organic and inorganic solid acids were developed. However, traditional methods often suffer from some drawbacks such as poor selectivity, harsh conditions, are not atom economic, or are not environmentally friendly.

**Scheme 1:**
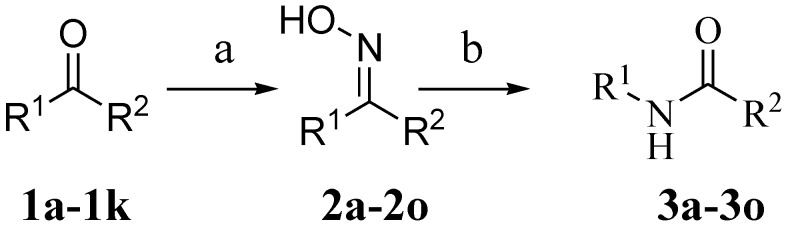
Synthesis of amides. Reagents and conditions: (**a**) NH_2_OH.HCl, NaOH, EtOH, H_2_O, reflux; (**b**) acidic ionic liquid, P_2_O_5_, N_2_, 90 °C, 6 h.

From the point of view of atom conversion efficiency, Beckmann rearrangement is a perfect way for construction of amides, in general sulfuric acid is most commonly used rearrangement catalyst in commercial production of amides. However, it brings equipment corrosion and environmental pollution problems. Recently, ionic liquids [17] have emerged as potential green alternatives to organic solvents due to their unique properties of low volatility, high polarity, good thermal stability, and excellent solubility [18,19,20]. Further, there are more potential capabilities as effective catalysts and reagents [13], as chemical transformations have also been explored. In order to develop a green pathway of amide synthesis, we report here a Beckmann rearrangement reaction catalyzed by *N*-methyl-imidazolium hydrosulfate ([HMIm]HSO_4_) [21] under solvent free conditions (Scheme 1).

## 2. Results

Beckmann rearrangement of benzophenone oxime catalyzed by [HMIm]HSO_4_ was carried out at 120 °C over 6 h without any solvent, the desired product, benzanilide, was obtained in moderate yield (45%). The co-catalysts such as P_2_O_5_, FeCl_3_, ZnCl_2_, CuCl_2_.2H_2_O, and AlCl_3_ were investigated in this reaction system, the yield was improved significantly to 91% with P_2_O_5_. However, it has been shown in the literature that the conversion is around 20% only when P_2_O_5_ is used as the sole catalyst of Beckmann rearrangement [15]. When CuCl_2_.2H_2_O was added, the yield was reduced to 14%, and the reverse reaction of benzophenone oxime was observed, benzophenone was regenerated. The results are presented in Table 1. 

**Figure 1:**
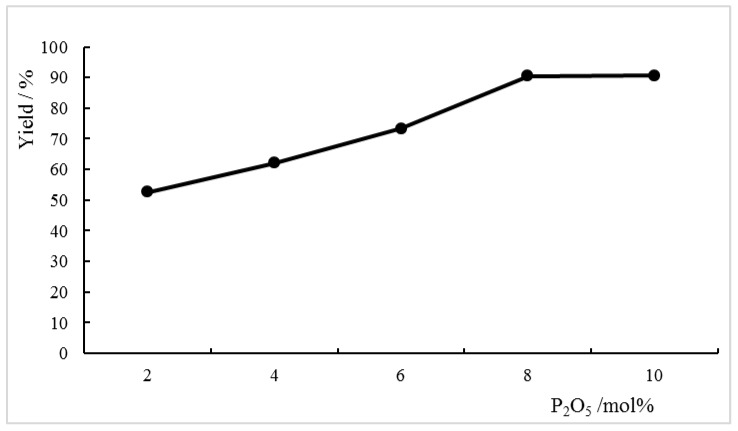
Effect of the amount of co-catalyst P_2_O_5_ on Beckmann rearrangement of benzophenone oxime. Reaction conditions: Benzophenone oxime (9.5 mmol), [HMIm]HSO_4_ (11.4 mmol), 90 °C, 6 h.

The effect of the amount of co-catalyst P_2_O_5_ on reaction was investigated. The reaction yield was improved when more co-catalyst was added, and the best yield was around 90% when the amount of P_2_O_5_ was higher than 8 mol %. The results are presented in Figure 1.

**Figure 2:**
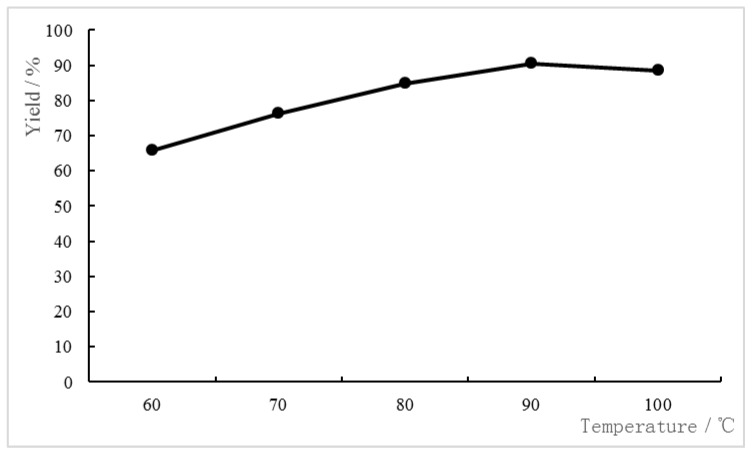
Influence of the reaction temperature on Beckmann rearrangement of benzophenone oxime. Reaction conditions: Benzophenone oxime (9.5 mmol), [HMIm]HSO_4_ (11.4 mmol), 6 h, co-catalyst (P_2_O_5_ 8%).

The influence of the reaction temperature on the yield was investigated subsequently. It was found that 90 °C is the best reaction temperature. The results are presented in Figure 2.

The recycling performance of ionic liquid has the most benefits from the point of view of environmental protection. During the reaction workup, the white product was precipitated out when ice water was added, after filtration, the mother liquid was evaporated in a vacuum, and ionic liquid was recovered and could be reused for three times. The results are presented in Table 2.

In order to explore the scope and limitations of this reaction, we extended the procedure to various aryl-substituted and alkyl-substituted ketoximes. In general, the reaction proceeded easily under the best conditions and the amide products were isolated in excellent yields and high purity. The results are presented in Table 3.

## 3. Experimental Section

All melting points were determined using a YRT-3 Digital Melting Point Apparatus (Tianjin, China). All melting points were uncorrected. All new compounds were characterized by HRMS-EI(M+), ^1^H and ^13^C-NMR spectra were recorded in CDCl_3_ or DMSO-d_6_ on a Bruker AV 600 MHz or Bruker AV 400 MHz instrument. HRMS spectra were obtained on an Agilent 6230 mass spectrometer.

### 3.1. Synthesis of N-methyl-imidazolium Hydrosulfate ([HMIm]HSO_4_)

*N*-Methylimidazole (8.2 g, 0.10 mol) was cooled down to 0 °C and concentrated sulfuric acid (10.0 g) was added dropwise. After addition, the solution was stirred 24 h at room temperature, a transparent viscous liquid (17.6 g) was obtained. Yield: 99%; IR (cm^−1^): 3345, 3150, 2870, 1447, 1337, 1221, 1048, 1082, 887.

### 3.2. General Procedures for Synthesis of Oxime Substrates **2a**–**2o**

Ketone (0.027 mol) and hydroxylamine hydrochloride (3.0 g, 0.043 mol) were dissolved in EtOH (10 mL) and H_2_O (20 mL). To the mixture was added NaOH (5.5 g, 0.137 mol). The reaction mixture was heated under reflux and the reaction was monitored by thin layer chromatography (TLC). After completion of the reaction, the reaction mixture was cooled down to room temperature, to the reaction mixture were added concentrated hydrochloric acid (15 mL) and water (100 mL). The solid was filtered off and recrystallized from EtOH, affording the products **2a**–**2o**.

**2a**: White solid, Yield: 91%. m.p.: 87.6–88.7 °C; ^1^H-NMR (400 MHz, CDCl_3_) δ 7.60 (d, *J* = 8.9 Hz, 2H), 6.93 (d, *J* = 8.9 Hz, 2H), 3.85 (s, 3H), 2.30 (s, 3H); ^1^^3^C-NMR (100 MHz, CDCl_3_) δ 160.5, 155.6, 129.0, 127.4, 113.9, 55.3, 13.3.

**2b**: White solid, Yield: 93%. m.p.: 88.0–89.0 °C; ^1^H-NMR (400 MHz, CDCl_3_) δ 7.55 (d, *J* = 8.2 Hz, 2H), 7.22 (d, *J* = 8.0 Hz, 2H), 2.39(s, 3H), 2.31 (s, 3H); ^13^C-NMR (100 MHz, CDCl_3_) δ 156.0, 139.3, 133.7, 129.3, 126.0, 21.3, 12.3.

**2c**: White solid, Yield: 90%. m.p.: 51.7–53.0 °C; ^1^H-NMR (600 MHz, CDCl_3_) δ 7.68–7.64 (m, 2H), 7.44–7.40 (m, 3H), 2.35(s, 3H); ^13^C-NMR (150 MHz, CDCl_3_) δ 156.1, 136.5, 129.3, 128.5, 126.1, 12.3.

**2d**: White solid, Yield: 70%. m.p.: 74.0–76.0 °C;^1^H-NMR (600 MHz, DMSO-*d*_6_) δ 11.21 (s, 1H), 7.76–7.59 (m, 2H), 7.22–7.19 (m, 2H), 2.14 (s, 3H); ^13^C-NMR (150 MHz, DMSO-*d*_6_) δ 162.4 (d, ^1^*J*_CF_ = 245.5 Hz), 152.1, 133.5 (d, ^4^*J*_CF_ = 3.0 Hz), 127.6 (d, ^3^*J*_CF_ = 8.3 Hz), 115.2 (d, ^2^*J*_CF_ = 21.5 Hz), 11.9.

**2e**: White solid, Yield: 91%. m.p.: 141.0–142.0 °C; ^1^H-NMR (600 MHz, DMSO-*d*_6_) δ 11.40 (s, 1H), 7.80 (t, *J* = 1.8 Hz, 1H), 7.64 (d, *J* = 7.8 Hz, 1H), 7.55 (d, *J* = 7.8 Hz, 1H), 7.34 (dd, *J* = 11.1, 4.6 Hz, 1H), 2.14 (s, 3H); ^13^C-NMR (150 MHz,DMSO-*d*_6_) δ 151.9, 139.3, 131.3, 130.6, 128.1, 124.6, 121.8, 11.4.

**2f**: Yellow solid, Yield: 90%. m.p.: 172.0–173.0 °C; ^1^H-NMR (400 MHz, DMSO-*d*_6_) δ 11.78 (s, 1H), 8.28—8.18 (m, 2H), 7.96–7.88 (m, 2H), 2.21 (s, 3H); ^13^C-NMR (100 MHz, DMSO-*d*_6_) δ 157.1, 152.5, 148.3, 131.9, 131.8, 16.6.

**2g**: White solid, Yield: 95.6%. m.p.: 129.6–131.0 °C; ^1^H-NMR (600 MHz, DMSO-*d*_6_) δ 10.49 (s, 1H), 7.42–7.27 (m, 5H), 3.04–2.85 (m, 1H), 1.75–1.66 (m, 2H), 1.64–1.50 (m, 6H); ^13^C-NMR (150 MHz, DMSO-*d*_6_) δ 158.6, 135.8, 128.3, 128.2, 128.1, 45.2, 30.3, 24.9.

**2h**: White solid, Yield: 60%. m.p.: 92.0–93.0 °C; ^1^H-NMR (600 MHz, DMSO-*d*_6_) δ 11.40 (s, 1H), 7.48–7.44 (m, 2H), 7.43–7.40 (m, 1H), 7.387.37 (m, 5H), 7.30–7.25 (m, 2H); ^13^C-NMR (150 MHz, DMSO-*d*_6_) δ 155.2, 136.8, 133.5, 128.88, 128.86, 128.40, 128.36, 128.2, 127.0.

**2i**: White solid, Yield: 87.5%. m.p.: 134.0–136.0 °C; ^1^H-NMR (600 MHz, CDCl_3_) δ 7.48 (d, *J* = 8.6 Hz, 2H), 7.43–7.37 (m, 4H), 7.36–7.31 (m, 2H); ^13^C-NMR (150 MHz, CDCl_3_) δ 156.1, 135.9, 135.5, 134.3, 130.8, 130.4, 129.1, 128.8, 128.7.

**2j**: White solid, Yield: 88.5%. m.p.: 131.0–132.0 °C; ^1^H-NMR (600 MHz, DMSO-*d*_6_) δ 11.45 (s, 1H), 7.43–7.38 (m, 2H), 7.38–7.32 (m, 2H), 7.28 (dd, *J* = 12.3, 5.4 Hz, 2H), 7.19 (dd, *J* = 12.3, 5.4 Hz, 2H); ^13^C-NMR (150 MHz, DMSO-*d*_6_) δ 162.6 (d, ^1^*J*_CF_ = 244.6 Hz), 161.9 (d, ^1^*J*_CF_ = 244.6 Hz), 153.4, 133.2, 131.3 (d, ^3^*J*_CF_ = 8.2 Hz), 129.5, 129.1 (d, ^3^*J_CF_* = 8.2 Hz), 115.4 (d, ^2^*J*_CF_ = 22.6 Hz), 115.2 (d, ^2^*J*_CF_ = 22.3 Hz).

**2k**: White solid, Yield: 87.5%. m.p.: 129.0–130.0 °C; ^1^H-NMR (400 MHz, DMSO-*d*_6_) δ 11.1 (s, 1H), 7.31 (d, *J* = 8.8 Hz, 2H), 7.24 (d, *J* = 8.8 Hz, 2H), 6.99 (d, *J* = 8.7 Hz, 2H), 6.91 (d, *J* = 8.8 Hz, 2H), 3.79 (s, 3H), 3.76 (s, 3H); ^13^C-NMR (100 MHz, DMSO-*d*_6_) δ 159.8, 159.1, 154.4, 130.7, 129.7, 128.6, 125.6, 113.7, 113.4, 55.2, 55.1.

**2l:** Light yellow solid, Yield: 87.9%. m.p.:155.0–160.0 °C; *(isomer 1)*: ^1^H-NMR (600 MHz, DMSO-*d*_6_) δ 11.27(s, 1H), 7.38–7.34 (m, 4H), 7.29–7.24 (m, 3H), 7.00 (d, *J* = 8.7 Hz, 2H), 3.79 (s, 3H); *(isomer 2)*: ^1^H-NMR (600 MHz, DMSO-*d*_6_) δ 11.09 (s, 1H), 7.46–7.43 (m, 2H), 7.42–7.38 (m, 3H), 7.30 (d, *J* = 8.8 Hz, 2H), 6.92 (d, *J* = 8.8 Hz, 2H), 3.75 (s, 3H); *(isomer 1)*: ^13^C-NMR (100 MHz, DMSO-*d_6_*) δ 159.85, 154.85, 137.32, 130.74, 129.22, 128.32, 128.29, 128.12, 113.80, 55.20; *(isomer 2)*: ^13^C-NMR (100 MHz, DMSO-*d_6_*) δ 159.20, 154.80, 133.82, 128.87, 128.76, 128.29, 127.32, 125.37, 113.46, 55.15.

**2m**: Yield: 94.0%. m.p.: 84–86 °C; ^1^H-NMR (600 MHz, DMSO-*d*_6_) δ 8.81 (brs, 1H), 7.30–7.18 (m, 5H), 2.83 (t, *J* = 8.2 Hz, 2H), 2.51 (t, *J* = 8.2 Hz, 2H), 1.91 (s, 3H); ^13^C-NMR (150 MHz, DMSO-*d*_6_) δ 157.9, 141.0, 128.4, 128.3, 126.1, 37.7, 32.6,13.8.

**2n**: Yield: 90.0%. m.p.: 79–81 °C; ^1^H-NMR (600 MHz, DMSO-*d*_6_) δ 12.74 (s, 1H), 7.73–7.16 (m, 5H); ^13^C-NMR (150 MHz, DMSO-*d*_6_) δ145.2 (q, ^2^*J*_CF_ = 30.0), 130.6, 129.0, 128.9, 127.2, 121.6 (q, ^1^*J*_CF3_ = 271.5).

**2o**: Yield: 85.5%. m.p.: 89–91 °C; ^1^H-NMR (600 MHz, DMSO-*d*_6_) δ 2.48 (dd, *J* = 6.8, 5.3, 2H), 2.48 (m, 2H), 1.76–1.45 (m, 6H); ^13^C-NMR (150 MHz, DMSO-*d*_6_) δ 157.1, 31.6, 26.6, 25.4, 25.2, 23.8.

### 3.3. General Procedures for the Synthesis of Amides **3a**–**3o**

To a solution of the oxime substrates **2a**–**2o** (9.50 mmol) in (HMIm)HSO_4_ (2.05 g, 11.4 mmol), the co-catalyst P_2_O_5_ (0.15 g, 1.0 mmol) was added. Then the solution was heated to 90 °C and the reaction was monitored by TLC. After completion of the reaction, the mixture was extracted with ethyl acetate (50 mL) twice, and the combined organic phase was washed with the aqueous solution of sodium bicarbonate and brine, dried over anhydrous Na_2_SO_4_ and concentrated in vacuo to afford a residue, which was purified by column (ethyl acetate: petroleum ether = 1:4) to afford the products **3a**–**3o**.

**3a [22]**: White solid, Yield: 91%. m.p.: 127.0–128.0 °C; ^1^H-NMR (600 MHz, DMSO-*d*_6_) δ 9.80 (brs, 1H), 7.51–7.46 (m, 2H), 7.20 (d, *J* = 7.9 Hz, 1H), 6.89–6.84 (m, 2H), 3.71 (s, 3H), 2.01 (s, 3H); ^13^C-NMR (150 MHz, DMSO-*d*_6_) δ 168.2, 155.5, 133.0, 121.0, 114.2, 55.6, 24.3; HRMS(+): calcd. for C_9_H_11_NO_2_ [M + H]^+^ 166.0863, found 166.0859; calcd. for C_9_H_11_NO_2_Na [M + Na]^+^ 188.0682, found 188.0682.

**3b [22]**: White solid, Yield: 90%. m.p.: 149.0–150.0 °C; ^1^H-NMR (600 MHz, DMSO-*d*_6_) δ 9.84 (s, 1H), 7.46 (d, *J* = 8.3 Hz, 2H), 7.09 (d, *J* = 8.3 Hz, 2H), 2.24 (s, 3H), 2.02 (s, 3H); ^13^C-NMR (150 MHz, DMSO-*d*_6_) δ 173.2, 142.1, 137.0, 134.1, 124.2, 29.2, 25.6; HRMS(+): calcd. for C_9_H_11_NO [M + H]^+^ 150.0913, found 150.0912; calcd. for C_9_H_11_NONa [M + Na]^+^ 172.0733, found 172.0741.

**3c [23]**: White solid, Yield: 90%. m.p.: 108.5–110.0 °C; ^1^H-NMR (600 MHz, DMSO-*d*_6_) δ 9.93 (brs, 1H), 7.58 (dd, *J* = 1.0, 8.5 Hz, 2H), 7.29 (dd, *J* = 7.5, 8.4 Hz, 2H), 7.08–6.91 (m, 1H), 2.05 (s, 3H); ^13^C-NMR (150 MHz, DMSO-*d*_6_) δ 168.7, 139.8, 129.1, 123.4, 119.4, 24.5; HRMS(+): calcd. for C_8_H_9_NO [M + H]^+^ 136.0757, found 136.0755; calcd. for C_8_H_9_NONa [M + Na]^+^ 158.0576, found 158.0572.

**3d [24]**: Light yellow solid, Yield: 89%. m.p.: 153–155 °C; ^1^H-NMR (600 MHz, DMSO-*d*_6_) δ 9.99 (brs, 1H), 7.87–7.47 (m, 2H), 7.12 (t, *J* = 8.99 Hz, 2H), 2.04 (s, 3H); ^13^C-NMR (150 MHz, DMSO-*d*_6_) δ 168.6, 158.3.8 (d, ^1^*J*_CF_ = 237.0 Hz), 136.2 (d, ^4^*J*_CF_ = 1.5 Hz), 121.1 (d,^3^*J*_CF_ = 7.5 Hz), 115.2 (d, ^2^*J*_CF_ = 22.5 Hz), 24.3; HRMS(+): calcd. for C_8_H_8_FNO [M + H]^+^ 154.0663, found 154.0665; calcd. for C_8_H_8_FNONa [M + Na]^+^ 176.0482, found 176.0481.

**3e [25]**: White solid, Yield: 88%. m.p.: 87.0–89.0 °C; ^1^H-NMR (600 MHz, DMSO-*d*_6_) δ 10.11 (brs, 1H), 7.95 (s, 1H), 7.47 (d, *J* = 7.9 Hz, 1H), 7.27–7.23 (m, 1H), 7.22–7.19 (m, 1H), 2.05 (s, 3H); ^13^C-NMR (150 MHz, CDCl_3_) δ 169.1, 141.3, 131.1, 126.0, 122.0, 121.7, 118.1, 24.5; HRMS(+): calcd. for C_8_H_8_BrNO [M + H]^+^ 213.9862, found 213.9860; calcd. for C_8_H_8_BrNONa [M + Na]^+^ 235.9681, found 235.9681.

**3f [25]**: Yellow solid, Yield: 86%. m.p.: 214.0–215.0 °C; ^1^H-NMR (600 MHz, DMSO-*d*_6_) δ 10.57 (s, 1H), 8.21 (d, *J* = 9.2 Hz, 2H), 7.82 (d, *J* = 9.4 Hz, 2H), 2.12 (s, 3H); ^13^C-NMR (150 MHz, DMSO-*d*_6_) δ 169.8, 145.9, 142.3, 125.4, 119.0, 24.7; HRMS(+): calcd. for C_8_H_8_N_2_O_3_ [M + H]^+^ 181.0608, found181.0610; Calcd. for C_8_H_8_N_2_O_3_Na [M + Na]^+^ 203.0433, found 203.0437.

**3g**: White solid, Yield: 91%. m.p.: 160.0–161.0 °C; ^1^H-NMR (600 MHz, DMSO-*d*_6_) δ 8.29 (d, *J* = 7.0 Hz, 1H), 7.88–7.80 (m, 2H), 7.54–7.49 (m, 1H), 7.48–7.42 (m, 2H), 4.46–3.96 (m, 1H), 1.96–1.81 (m, 2H), 1.74–1.64 (m, 2H), 1.60–1.43 (m, 4H); ^13^C-NMR (150 MHz, DMSO-*d*_6_) δ 166.4, 135.3, 131.4, 128.6, 127.7, 51.4, 32.6, 24.1; HRMS(+): calcd. for C_12_H_15_NO [M + H]^+^ 190.1226, found 190.1228; calcd. for C_12_H_15_NO Na [M + Na]^+^ 212.1046, found 212.1044.

**3h [23]**: White solid, Yield: 89%. m.p.: 162.6–163.0 °C; ^1^H-NMR (400 MHz, DMSO-*d*_6_) δ 10.25 (s, 1H), 7.99–7.92 (m, 2H), 7.78 (d, *J* = 7.6 Hz, 2H), 7.63–7.58 (m, 1H), 7.57–7.51 (m, 2H), 7.36 (t, *J* = 7.9 Hz, 2H), 7.15–7.07 (m, 1H); ^13^C-NMR (100 MHz, DMSO-*d*_6_) δ 166.0, 139.6, 135.5, 132.0, 129.1, 128.9, 128.1, 124.1, 120.8; HRMS(+): calcd. for C_13_H_11_NO [M + H]^+^ 198.0913, found 198.0913; calcd. for C_13_H_11_NONa [M + Na]^+^ 220.0733, found 220.0733.

**3i [25]**: White solid, Yield: 85%. m.p.: 210.0–212.0 °C; ^1^H-NMR (600 MHz, DMSO-*d*_6_) δ 10.45 (s, 1H), 8.02–7.96 (m, 2H), 7.86–7.79 (m, 2H), 7.66–7.59 (m, 2H), 7.45–7.39 (m, 2H); ^13^C-NMR (150 MHz, DMSO-*d*_6_) δ 165.0, 138.4, 137.0, 133.8, 130.1, 129.0, 127.9, 122.4; HRMS(+): calcd. for C_13_H_9_C_l2_NO [M + H]^+^ 266.0134, found 266.0129; calcd. for C_13_H_9_C_l2_NONa [M + Na]^+^ 287.9953, found 287.9951.

**3j [26]**: Light yellow solid, Yield: 84%. m.p.: 183–185.0 °C; ^1^H-NMR (600 MHz, DMSO-*d*_6_) δ 10.31 (s, 1H), 8.04–8.02 (m, 2H), 7.82–7.72 (m, 2H), 7.37 (t, *J* = 8.8 Hz, 2H), 7.19 (t, *J* = 8.8 Hz, 2H); ^13^C-NMR (150 MHz, DMSO-*d*_6_) δ 164.3, 164.1 (d, ^1^*J*_CF_ = 249.5 Hz), 158.3 (d, ^1^*J*_CF_ = 240.3 Hz), 135.4, 131.2, 130.4 (d, ^3^*J*_CF_ = 9.0 Hz), 122.2 (d, ^3^*J*_CF_ = 7.8 Hz), 115.3 (d, ^2^*J*_CF_ = 22.4 Hz), 115.2 (d, ^2^*J*_CF_ = 22.8 Hz); HRMS(+): calcd. for C_13_H_9_F_2_NO [M + H]^+^ 234.0725, found 234.0727; calcd. for C_13_H_9_F_2_NONa [M + Na]^+^ 256.0544, found 256.0546.

**3k [26]**: Light yellow solid, Yield: 90%. m.p.: 204.0–205.0 °C; ^1^H-NMR (600 MHz, CDCl_3_) δ 7.83 (d, *J* = 8.8 Hz, 2H), 7.65 (s, 1H), 7.52 (d, *J =* 9.0 Hz, 2H), 6.97 (d, *J* = 8.8 Hz, 2H), 6.91 (d, *J* = 9.0 Hz, 2H), 3.87 (s, 3H), 3.81 (s, 3H); ^13^C-NMR (150 MHz, DMSO-*d*_6_) δ 164.5, 161.7, 155.4, 132.4, 129.4, 127.1, 121.9, 113.7, 113.5, 55.4, 55.2; HRMS(+): calcd. for C_15_H_15_NO_3_ [M + H]^+^ 258.1125, found 258.1127; calcd. for C_15_H_15_NO_3_Na [M + Na]^+^ 280.0944, found 280.0946.

**3l [25]** (*N-(4-Methoxyphenyl)benzamide*): Light yellow solid, Yield: 52%. M.p.: 156.5–159.5 °C; ^1^H-NMR (600 MHz, DMSO-*d*_6_) δ 10.07 (s, 1H), 7.95 (d, *J* = 9.2 Hz, 2H), 7.74 (d, *J* = 7.6 Hz, 2H), 7.51 (t, *J* = 7.6 Hz, 1H), 7.33 (t, *J* = 7.9 Hz, 2H), 7.05 (d, *J* = 9.2 Hz, 2H), 3.84 (s, 3H); ^13^C-NMR (100 MHz, DMSO-*d*_6_) δ 164.94, 161.91, 139.36, 131.40, 129.61, 128.58, 123.45, 120.37, 113.62, 55.45; HRMS(+): calcd. for C_14_H_13_NO_2_ [M + H]^+^ 228.1019, found 228.1020; calcd. for C_14_H_13_NO_2_Na [M + Na]^+^ 250.0838, found 250.0838; (*4-Methoxy-N-phenylbenzamide*): Light yellow solid, Yield: 28% ^1^H-NMR (600 MHz, DMSO-*d*_6_) δ 10.12 (s, 1H), 7.94 (d, *J* = 7.2 Hz, 2H), 7.67 (d, *J* = 9.0 Hz, 2H), 7.57 (t, *J* = 7.2 Hz, 1H), 7.09 (t, *J* = 8.8 Hz, 2H), 6.93 (d, *J* = 9.0 Hz, 2H), 3.74 (s, 3H); ^13^C-NMR (100 MHz, DMSO-*d*_6_) δ 165.14, 155.58, 135.07, 132.24, 128.37, 127.56, 127.00, 122.02, 113.76, 55.20. HRMS(+): calcd. for C_14_H_13_NO_2_ [M + H]^+^ 228.1019, found 228.1020; calcd. for C_14_H_13_NO_2_Na [M + Na]^+^ 250.0838, found 250.0838.

**3m [27]:** Yield: 89%. m.p.: 113–114 °C; ^1^H-NMR (600 MHz, DMSO-*d*_6_) δ 7.34–7.30 (m, 2H), 7.26–7.21 (m, 3H), 3.85–3.77 (m, 2H), 2.84–2.74 (m, 2H), 2.29 (s, 3H); ^13^C-NMR (150 MHz, DMSO-*d*_6_) δ 173.4, 139.0, 129.3, 129.0, 126.9, 46.4, 34.8, 26.6. HRMS(+): calcd. for C_10_H_13_NO [M + H]^+^ 164.1070, found 164.1071; calcd. for C_10_H_13_NONa [M + Na]^+^ 186.0889, found 186.0884.

**3n [28]:** Yield: 82%. m.p.: 84–87 °C; ^1^H-NMR (600 MHz, DMSO-*d*_6_) δ 11.26 (brs, 1H), 7.70 (dd, *J* = 0.83, 8.53 Hz, 2H), 7.43–7.38 (m, 2H), 7.24–7.20 (m, 1H), ^13^C-NMR (150 MHz, DMSO-*d*_6_) δ 155.0 (q, ^2^*J*_CF_ = 36.0), 136.8, 129.4, 126.0, 121.5, 116.3 (q, ^1^*J*_CF3_ = 286.5). HRMS(+): calcd. for C_8_H_6_F_3_NO [M + H]^+^ 190.0474, found 190.0472; calcd. for C_8_H_6_F_3_NONa [M + Na]^+^ 212.0294, found 212.0296.

**3o [29]:** Yield: 85%. m.p.: 68–71 °C; ^1^H-NMR (600 MHz, DMSO-*d*_6_) δ 7.41 (brs, 1H), 3.05 (dd, *J* = 5.87, 10.09 Hz, 2H), 2.38–2.13 (m, 2H), 1.66 (q, *J* = 5.87 Hz, 2H), 1.56–1.46 (m, 4H); ^13^C-NMR (150 MHz, DMSO-*d*_6_) δ 177.4, 41.9, 36.9, 30.5, 30.3, 23.4, HRMS(+): calcd. for C_6_H_11_NO [M + H]^+^ 114.0913, found 114.0910; calcd. for C_6_H_11_NONa [M + Na]^+^ 136.0733, found136.0734.

## 4. Conclusions

In conclusion, we successfully demonstrated an efficient approach for the synthesis of amide derivatives via Beckmann rearrangement of ketoxime by using Brønsted acidic ionic liquid *N*-methyl-imidazolium hydrosulfate as an environmental friendly catalyst and solvent. The best reaction condition is: reaction temperature 90 °C, reaction time 6 h, solvent *N*-methyl-imidazolium hydrosulfate 10 grams, co-catalyst P_2_O_5_ 8 mol %. Ionic liquid can be reused three times. The procedure can be extended to various symmetrical and unsymmetrical aryl-substituted and alkyl-substituted ketoxime substrates. The aryl group migration products are sole products for unsymmetrical aryl alkyl substituted amides. 

## Figures and Tables

**Table 1:** Effect of co-catalyst on Beckmann rearrangement in ionic liquid systems.

Entry	Co-Catalyst	Yield (%)
1	^a^	45
2	P_2_O_5_	91
3	FeCl_3_	47
4	AlCl_3_	50
5	ZnCl_2_	53
6	CuCl_2_·2H_2_O	14 ^b^

Reaction conditions: Benzophenone oxime (9.5 mmol), [HMIm]HSO_4_ (11.4 mmol), and co-catalyst (8%), 90 °C, 6 h; ^a^ without co-catalyst; ^b^ benzophenone(%) was obtained.

**Table 2:** Effect of ionic liquid recycling on Beckmann rearrangement.

Reaction Turn	P_2_O_5_/%	Conversion/%	Yield/%
1	8	91	91
2	1	91	90
3	0.5	90	88

Reaction conditions: Benzophenone oxime (9.5 mmol), [HMIm]HSO_4_ (11.4 mmol), 90 °C, 6 h; Conversion was determined by Gas Chromatography (GC) using internal standard method.

**Table 3:** Formation of amides (**3a**–**3o**) from ketoxime (**2a**–**2o**) in the presence of ionic liquid and co-catalyst P_2_O_5_.

Compd.	*R* ^1^	*R* ^2^	Yield %
**3a**	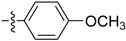	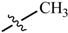	91
**3b**	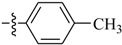	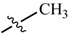	90
**3c**	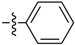	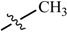	90
**3d**	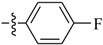	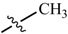	89
**3e**	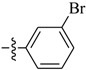	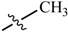	88
**3f**	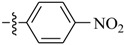	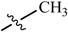	86
**3g**	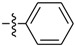	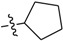	91
**3h**	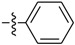	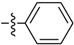	89
**3i**	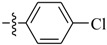	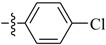	85
**3j**	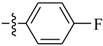	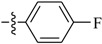	84
**3k**	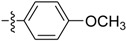	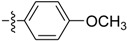	90
**3l(3l^’^)**	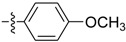	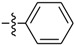	52
	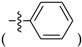	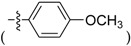	28
**3m**	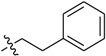	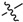	89
**3n**	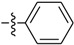	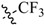	82
**3o**	Caprolactam		85

Reaction conditions: Benzophenone oxime (9.5 mmol), [HMIm]HSO_4_ (11.4 mmol), catalyst (P_2_O_5_ 8%), 90 °C, 6 h.

## Supplementary Materials

Supplementary File 1
