# Supplementary material for: Beckmann Rearrangement of Ketoxime Catalyzed by N-methyl-imidazolium Hydrosulfate
Source: Molecules. 2018 Jul 18;23(7):1764. doi: 10.3390/molecules23071764 (PMC6100402; doi:10.3390/molecules23071764)
Supplement: Supplementary File 1 [file molecules-23-01764-s001.pdf]

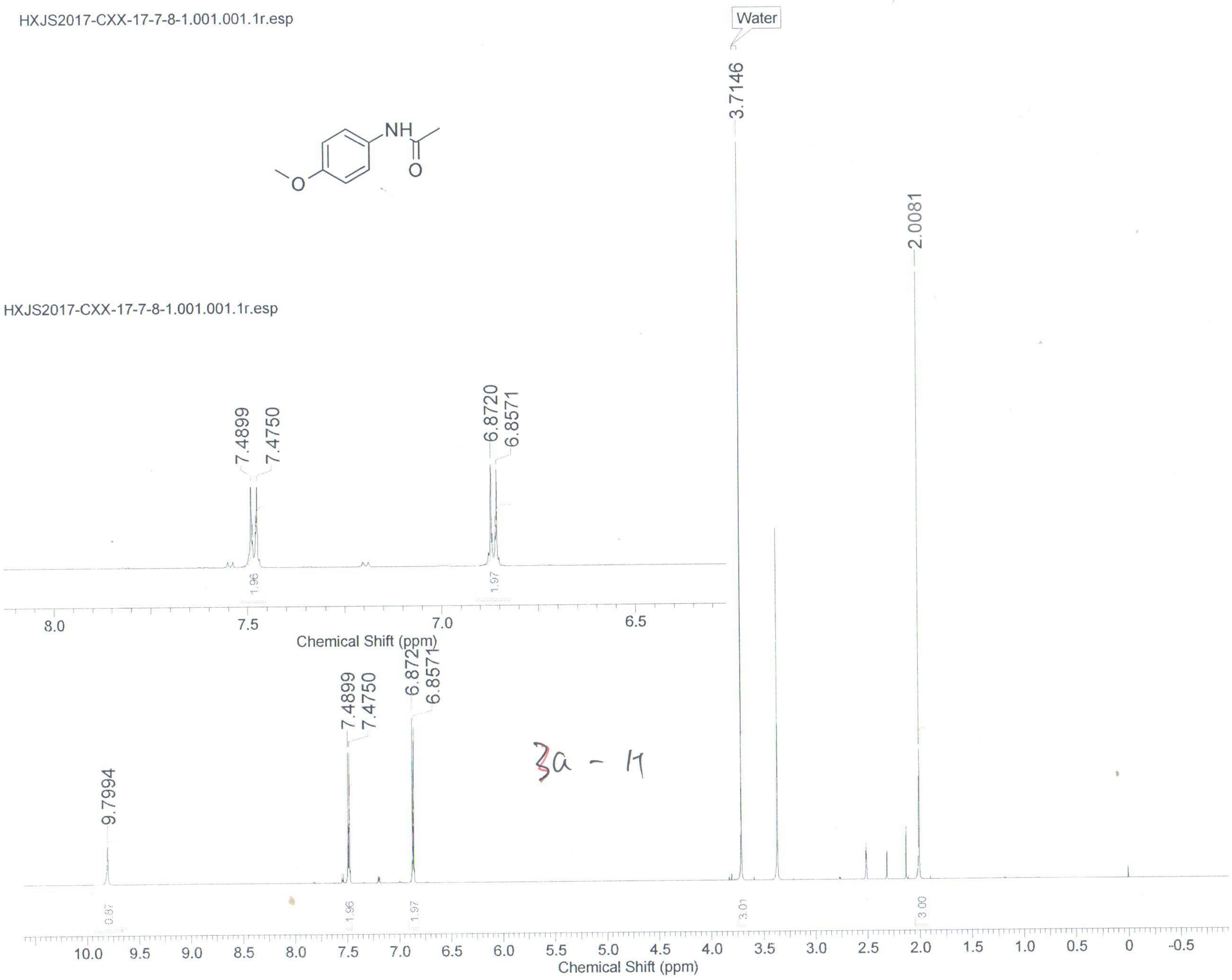

Acquisition Time (sec)	2.7263
Comment	1H NMR
Date	12 Jul 2017 09:23:12
Date Stamp	12 Jul 2017 09:23:12
Frequency (MHz)	600.13
Nucleus	1H
Number of Transients	4
Origin	spect
Original Points Count	32768
Owner	root
Points Count	65536
Pulse Sequence	zg30
Receiver Gain	78.68
SW(cyclical) (Hz)	12019.23
Solvent	DMSO-d6
Spectrum Offset (Hz)	3706.0515
Spectrum Type	STANDARD
Sweep Width (Hz)	12019.05
Temperature (degree C)	21.678

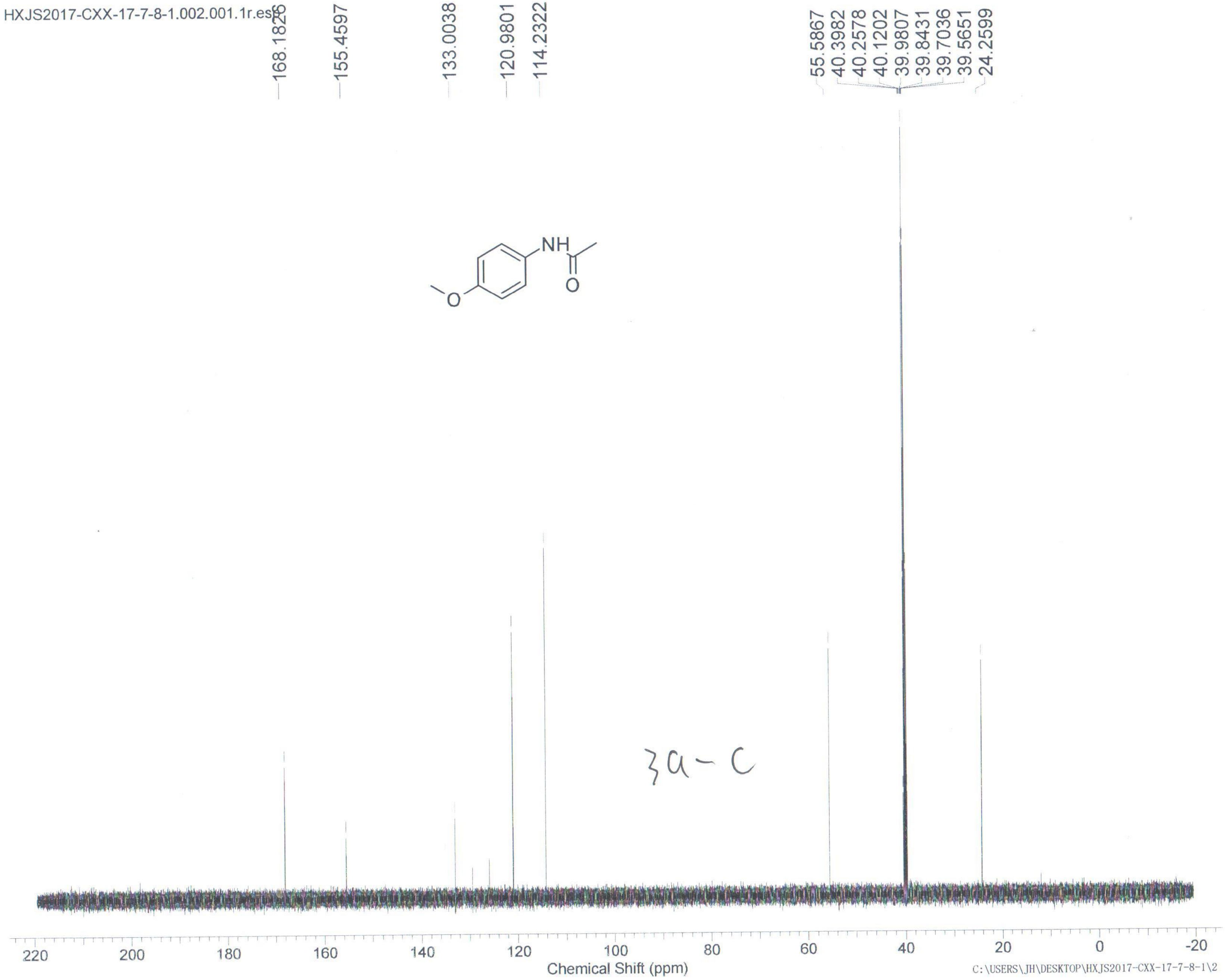

Acquisition Time (sec)	1.8175
Comment	13C NMR
Date	12 Jul 2017 09:27:28
Date Stamp	12 Jul 2017 09:27:28
Frequency (MHz)	150.90
Nucleus	13C
Number of Transients	100
Origin	spect
Original Points Count	65536
Owner	root
Points Count	262144
Pulse Sequence	zgpg30
Receiver Gain	197.01
SW(cyclical) (Hz)	36057.69
Solvent	DMSO-d6
Spectrum Offset (Hz)	15089.0967
Spectrum Type	STANDARD
Sweep Width (Hz)	36057.55
Temperature (degree C)	22.689

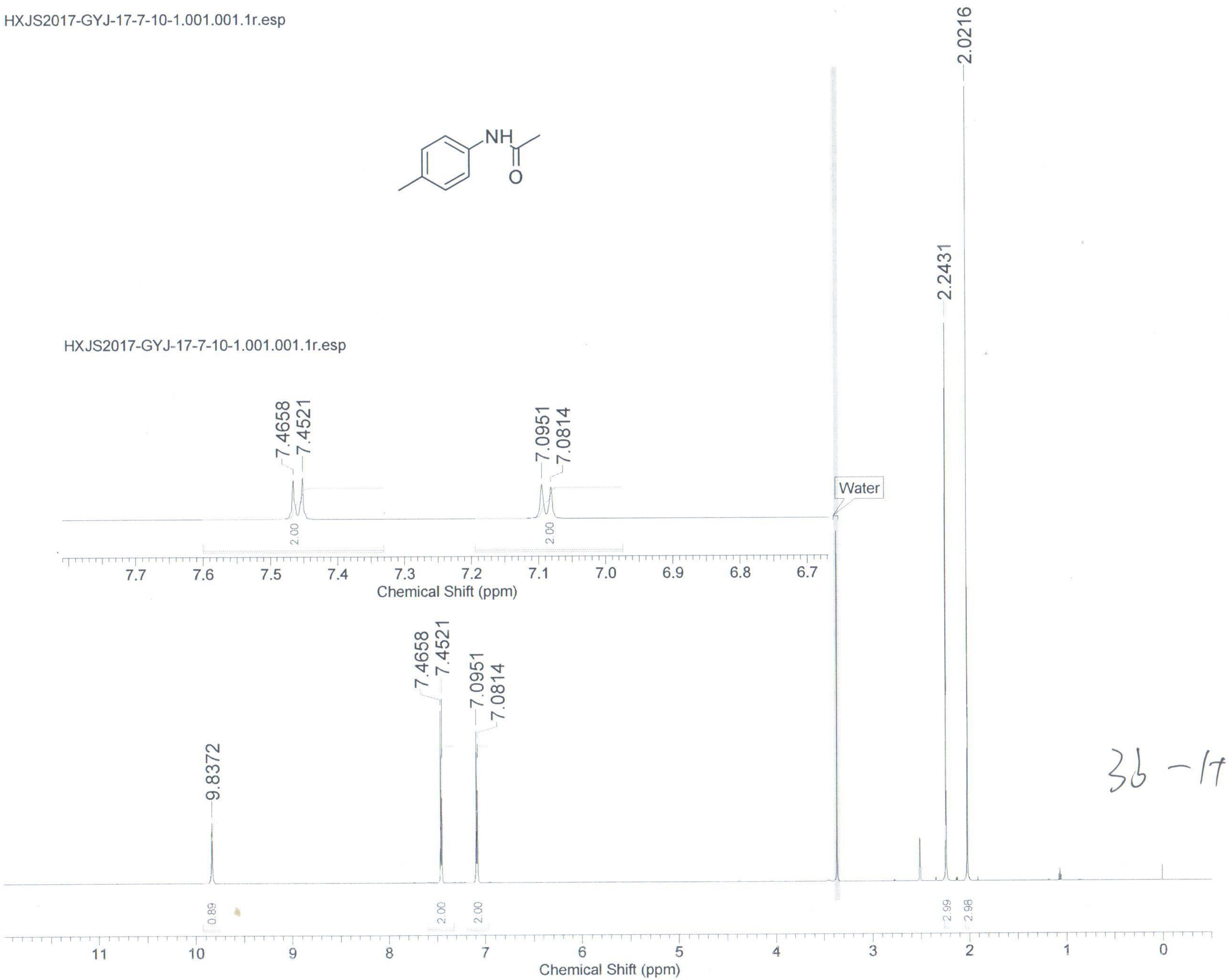

**Acquisition Time (sec)**	2.7263
**Comment**	1H NMR
**Date**	12 Jul 2017 09:18:56
**Date Stamp**	12 Jul 2017 09:18:56
**Frequency (MHz)**	600.13
**Nucleus**	1H
**Number of Transients**	4
**Origin**	spect
**Original Points Count**	32768
**Owner**	root
**Points Count**	65536
**Pulse Sequence**	zg30
**Receiver Gain**	78.68
**SW(cyclical) (Hz)**	12019.23
**Solvent**	DMSO-d6
**Spectrum Offset (Hz)**	3706.0515
**Spectrum Type**	STANDARD
**Sweep Width (Hz)**	12019.05
**Temperature (degree C)**	21.680

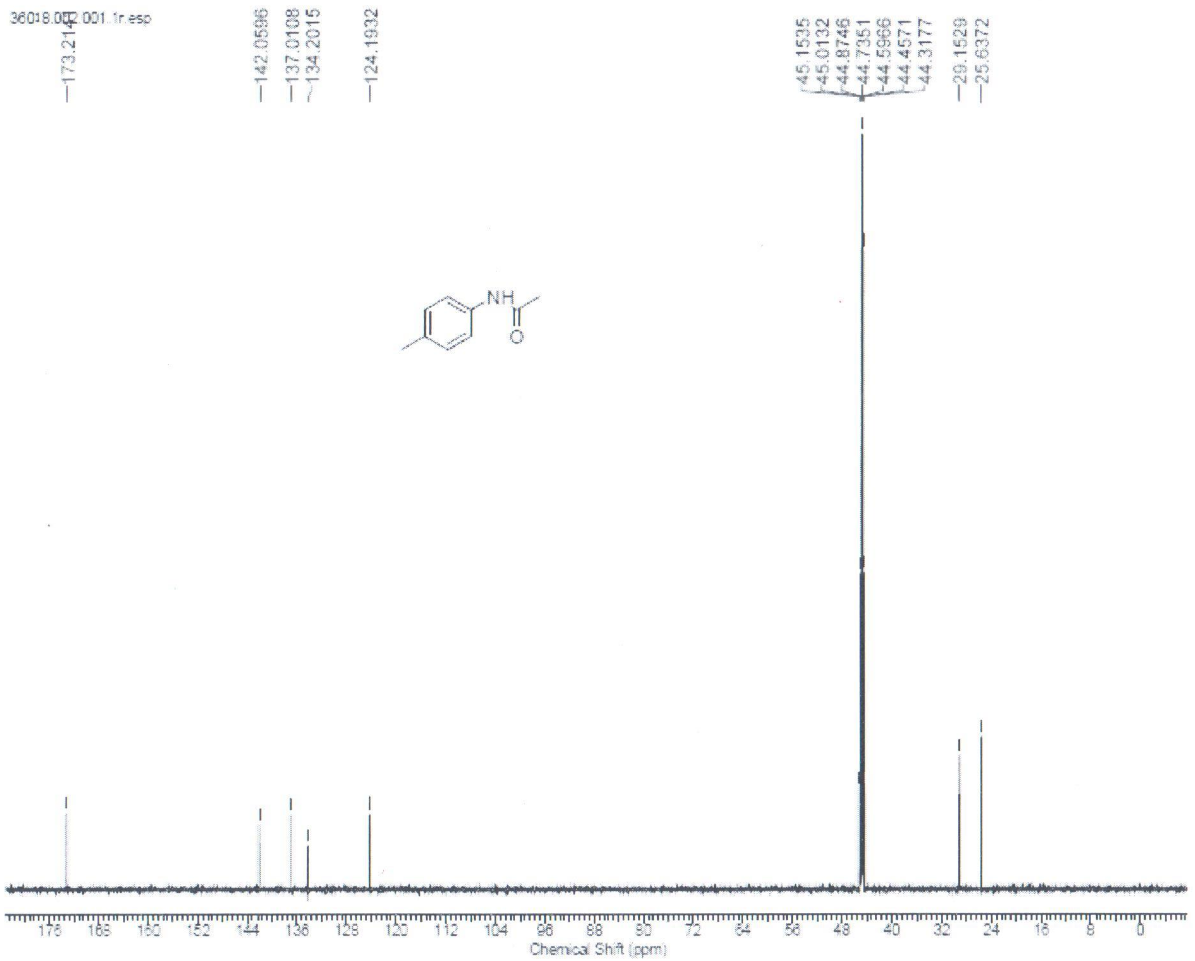

Acquisition Time (sec)	1.6175
Comment	13C NMR
Date	11 Jul 2017 16:29:52
Date Stamp	11 Jul 2017 16:29:52
Frequency (MHz)	150.90
Nucleus	13C
Number of Transients	100
Origin	spect
Original Points Count	65536
Owner	root
Points Count	262144
Pulse Sequence	zgpg30
Receiver Gain	197.01
SW(cyclical) (Hz)	36057.69
Solvent	CHLOROFORM-d
Spectrum Offset (Hz)	15089.0967
Spectrum Type	STANDARD
Sweep Width (Hz)	36057.55
Temperature (degree C)	23.085

3b-C

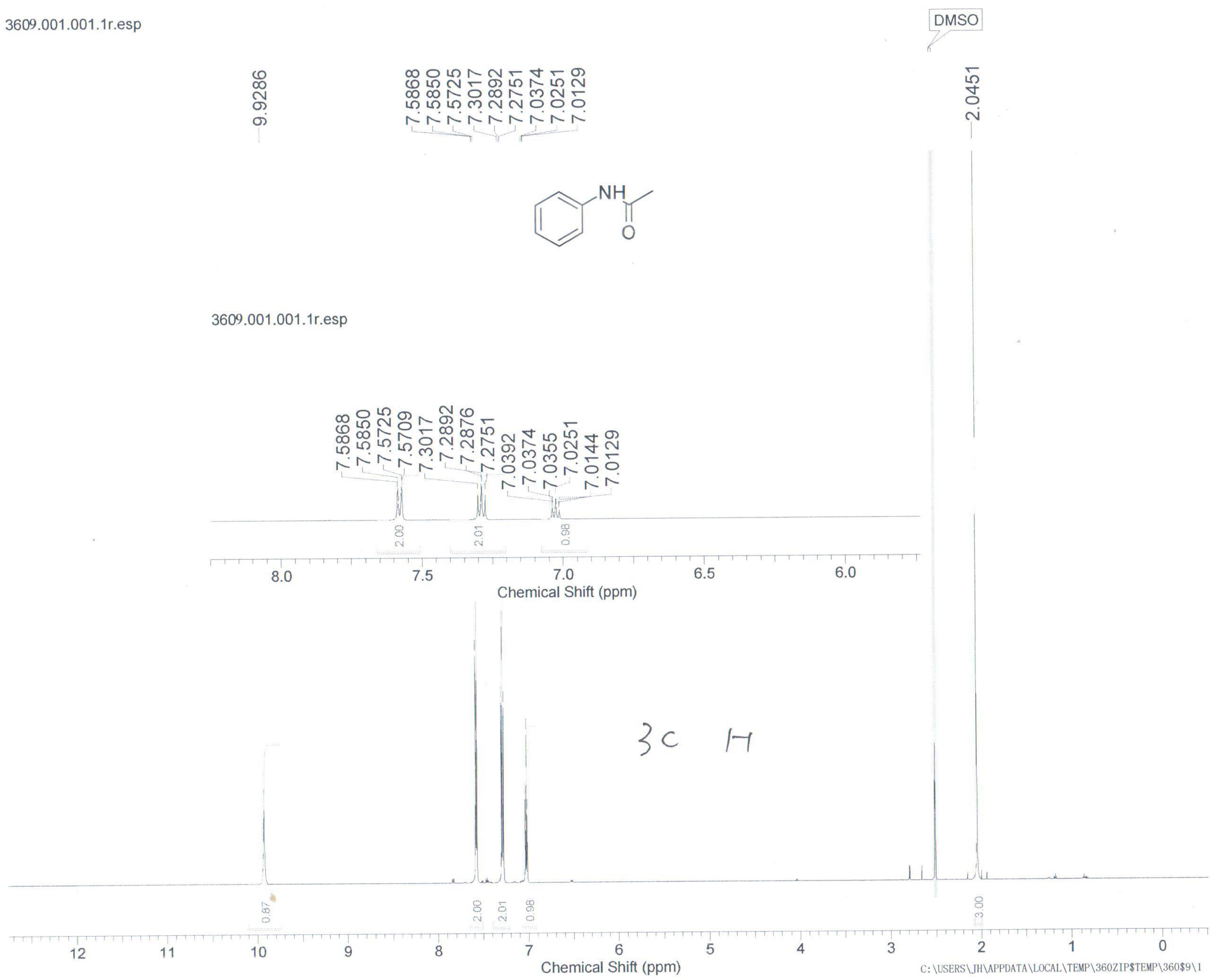

**Acquisition Time (sec)**	2.7263
**Comment**	1H NMR
**Date**	11 Jul 2017 16:44:48
**Date Stamp**	11 Jul 2017 16:44:48
**Frequency (MHz)**	600.13
**Nucleus**	1H
**Number of Transients**	4
**Origin**	spect
**Original Points Count**	32768
**Owner**	root
**Points Count**	65536
**Pulse Sequence**	zg30
**Receiver Gain**	78.68
**SW(cyclical) (Hz)**	12019.23
**Solvent**	DMSO-d6
**Spectrum Offset (Hz)**	3706.0515
**Spectrum Type**	STANDARD
**Sweep Width (Hz)**	12019.05
**Temperature (degree C)**	22.076

C:\USERS\JH\APPDATA\LOCAL\TEMP\360ZIP$TEMP\360$9\1

36010.002.001.1r.esp

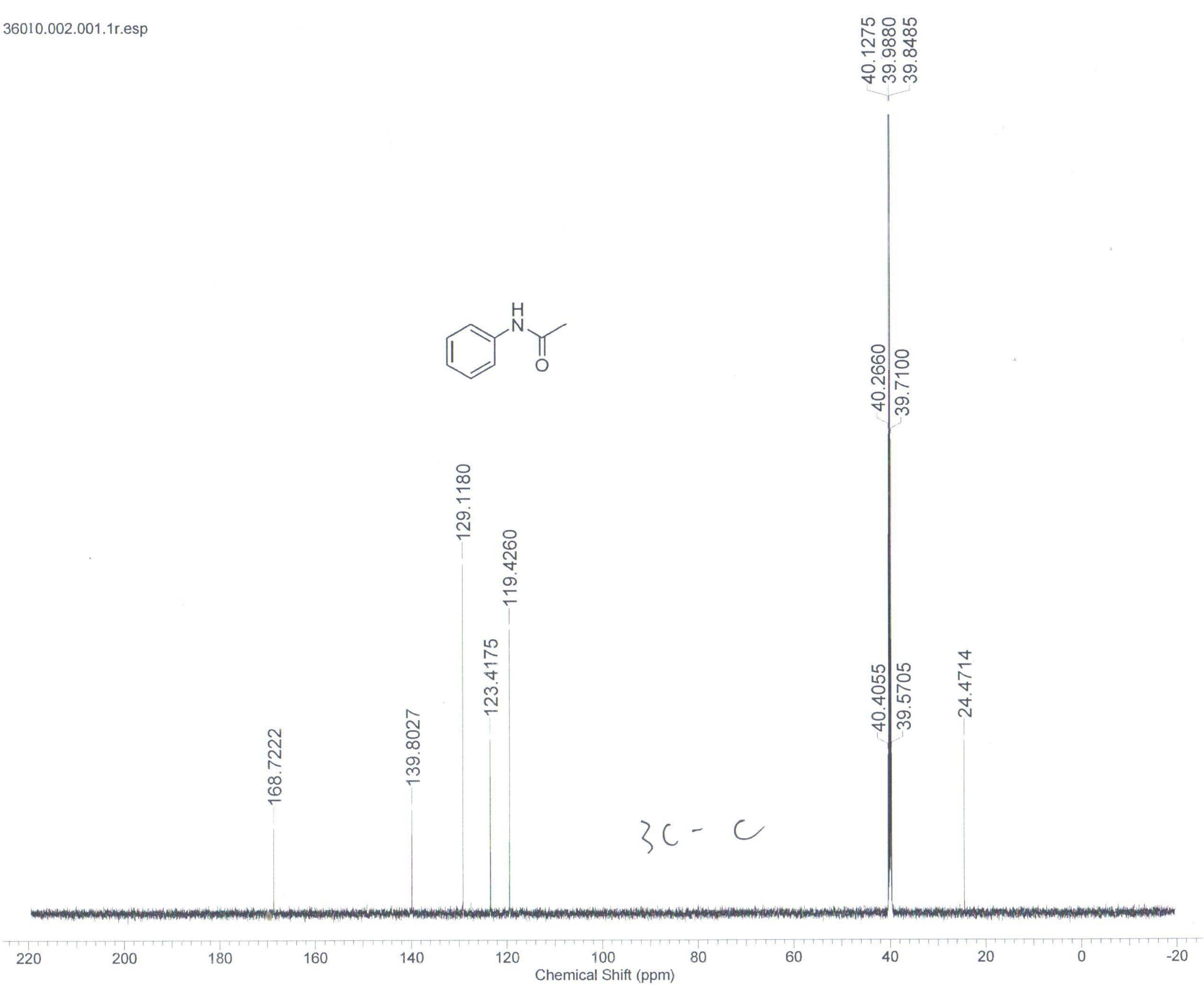

Acquisition Time (sec)	1.8175
Comment	13C NMR
Date	11 Jul 2017 16:40:32
Date Stamp	11 Jul 2017 16:40:32
Frequency (MHz)	150.90
Nucleus	13C
Number of Transients	65
Origin	spect
Original Points Count	65536
Owner	root
Points Count	262144
Pulse Sequence	zgpg30
Receiver Gain	197.01
SW(cyclical) (Hz)	36057.69
Solvent	DMSO-d6
Spectrum Offset (Hz)	15089.0967
Spectrum Type	STANDARD
Sweep Width (Hz)	36057.55
Temperature (degree C)	23.471

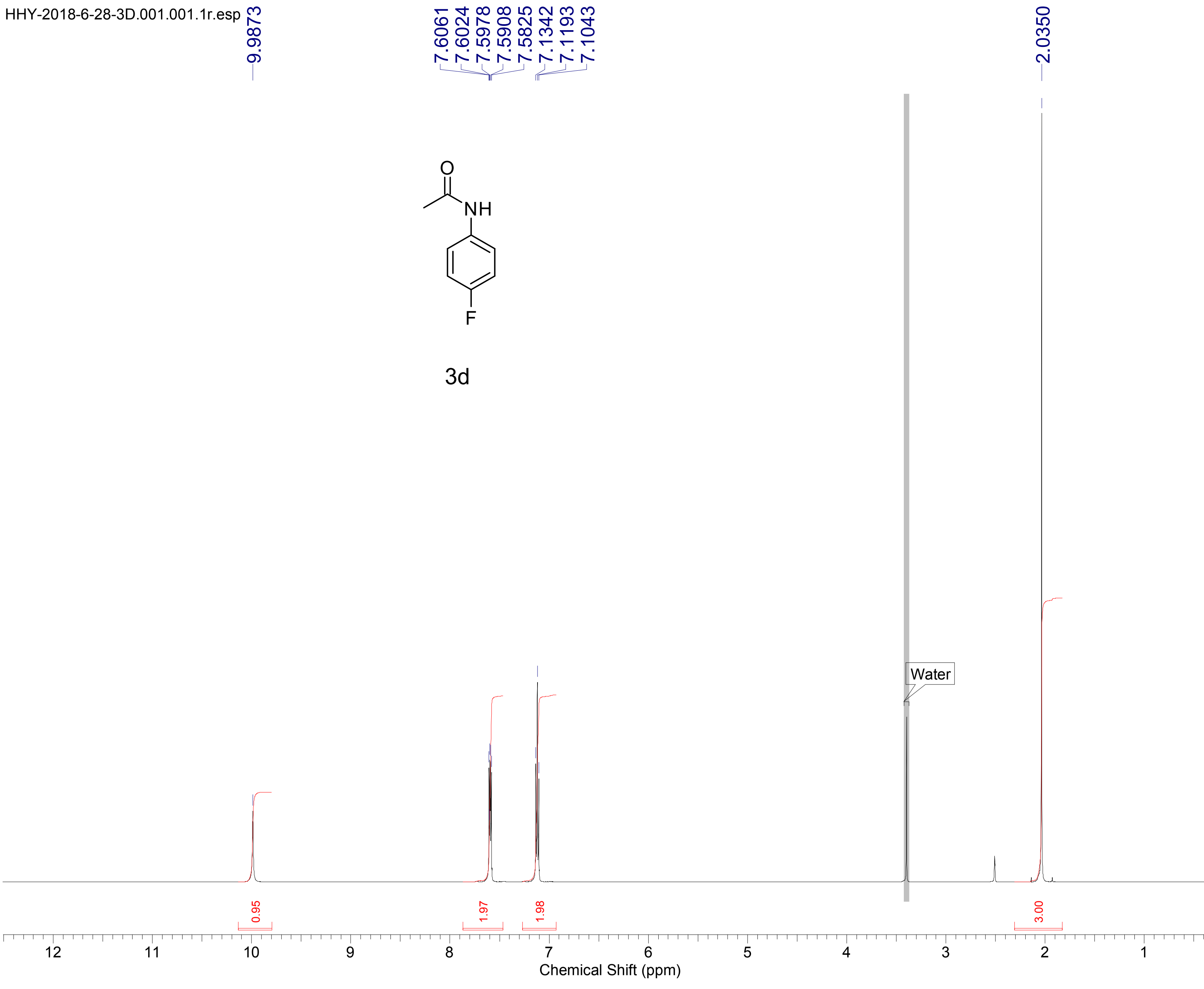
HHY-2018-6-28-3D.001.001.1r.esp
9.9873
7.6061
7.6024
7.5978
7.5908
7.5825
7.1342
7.1193
7.1043
2.0350
O
NH
F
3d
Water
0.95
1.97
1.98
3.00
12
11
10
9
8
7
6
5
4
3
2
1
Chemical Shift (ppm)

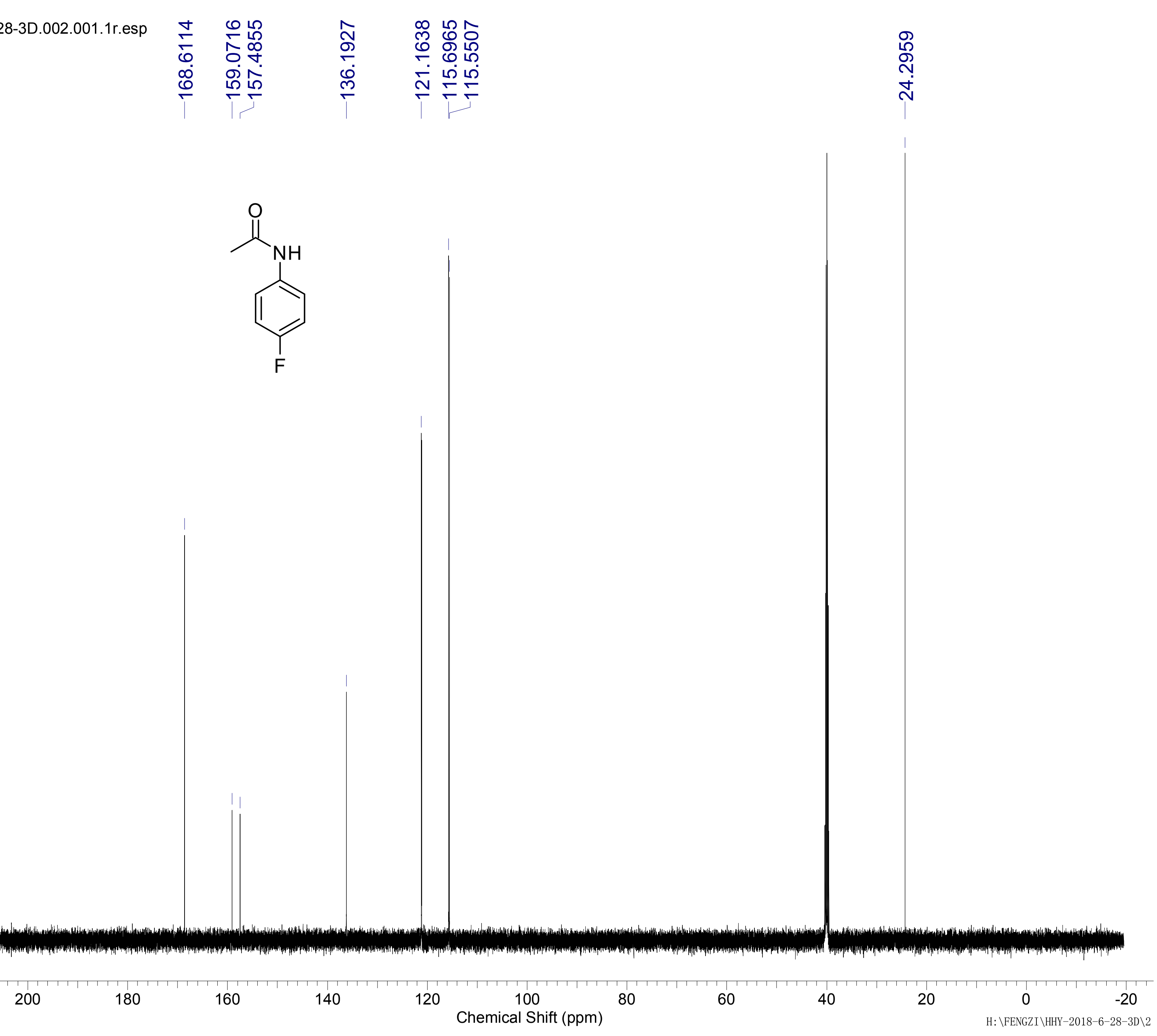

28-3D.002.001.1r.esp
168.6114
159.0716
157.4855
136.1927
121.1638
115.6965
115.5507
24.2959
O
NH
F
200
180
160
140
120
100
80
60
40
20
0
-20
Chemical Shift (ppm)
H:\FENGZI\HHY-2018-6-28-3D\2

HHY-2018-6-28-3E.001.001.1r.esp

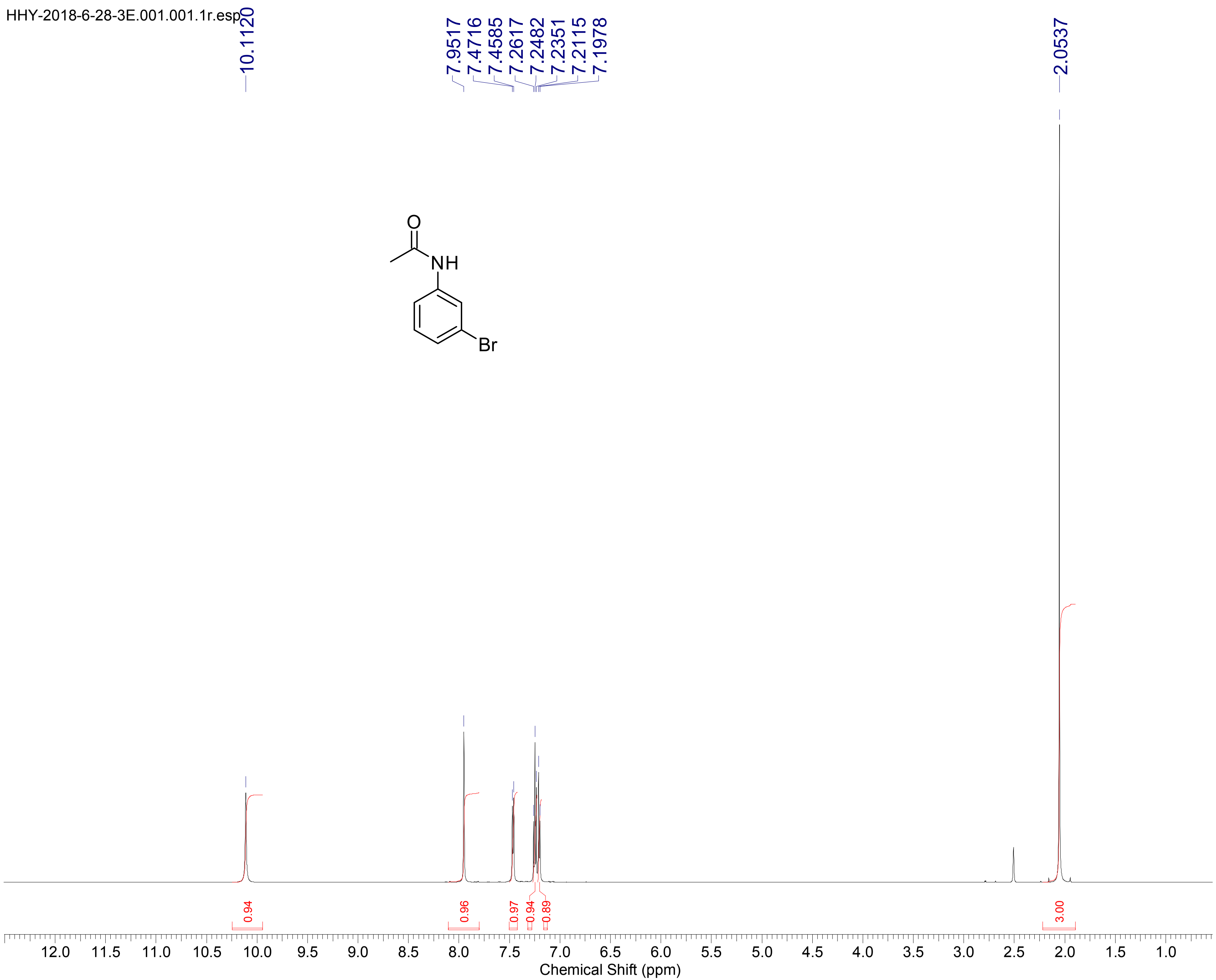

Acquisition Time (sec)	2.7263
Comment	1H NMR
Date	28 Jun 20[illegible] 16:19:12
Date Stamp	28 Jun 20[illegible] 16:19:12
Frequency (MHz)	600.13
Nucleus	1H
Number of Transients	8
Origin	spect
Original Points Count	32768
Owner	root
Points Count	65536
Pulse Sequence	zg30
Receiver Gain	43.49
SW(cyclical) (Hz)	12019.23
Solvent	DMSO-d6
Spectrum Offset (Hz)	3706.051
Spectrum Type	STANDA[illegible]
Sweep Width (Hz)	12019.05
Temperature (degree C)	22.348

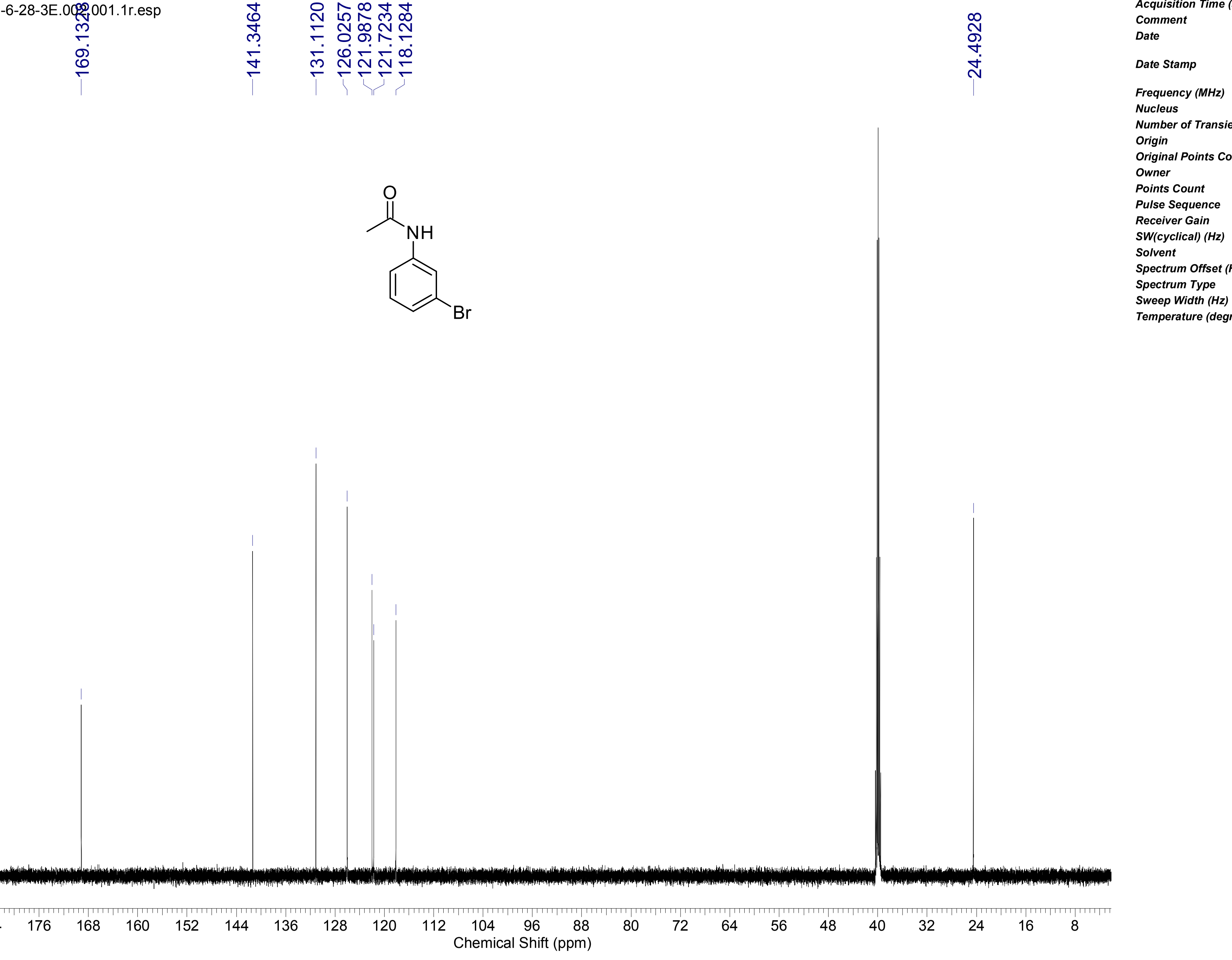
-6-28-3E.002.001.1r.esp
169.1328
141.3464
131.1120
126.0257
121.9878
121.7234
118.1284
24.4928
NH
O
Br
Acquisition Time (
Comment
Date
Date Stamp
Frequency (MHz)
Nucleus
Number of Transie
Origin
Original Points Co
Owner
Points Count
Pulse Sequence
Receiver Gain
SW(cyclical) (Hz)
Solvent
Spectrum Offset (
Spectrum Type
Sweep Width (Hz)
Temperature (degr
176
168
160
152
144
136
128
120
112
104
96
88
80
72
64
56
48
40
32
24
16
8
Chemical Shift (ppm)

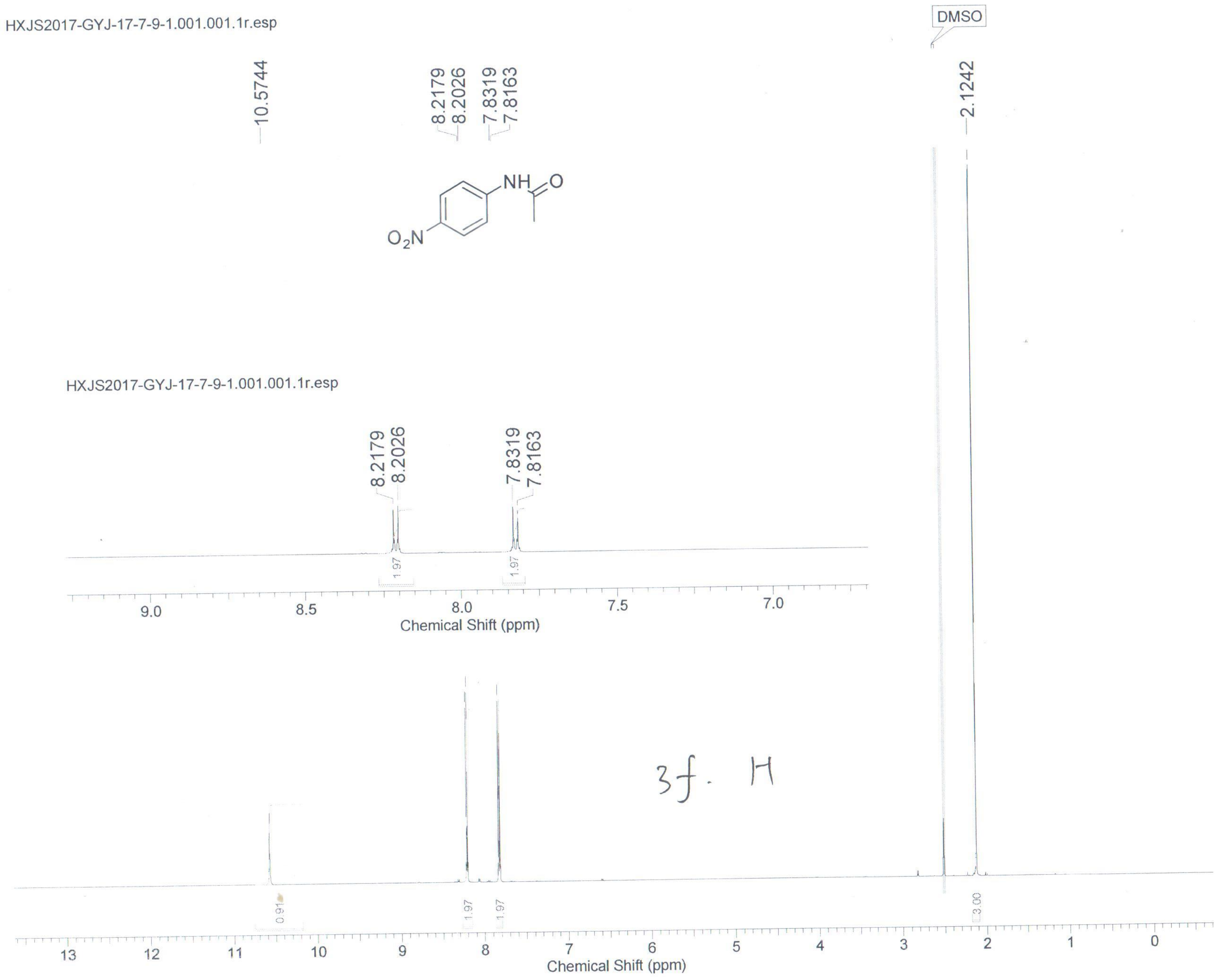

Acquisition Time (sec)	2.7263
Comment	1H NMR
Date	13 Jul 2017 11:09:52
Date Stamp	13 Jul 2017 11:09:52
Frequency (MHz)	600.13
Nucleus	1H
Number of Transients	4
Origin	spect
Original Points Count	32768
Owner	root
Points Count	65536
Pulse Sequence	zg30
Receiver Gain	78.68
SW(cyclical) (Hz)	12019.23
Solvent	DMSO-d6
Spectrum Offset (Hz)	3706.0515
Spectrum Type	STANDARD
Sweep Width (Hz)	12019.05
Temperature (degree C)	21.496

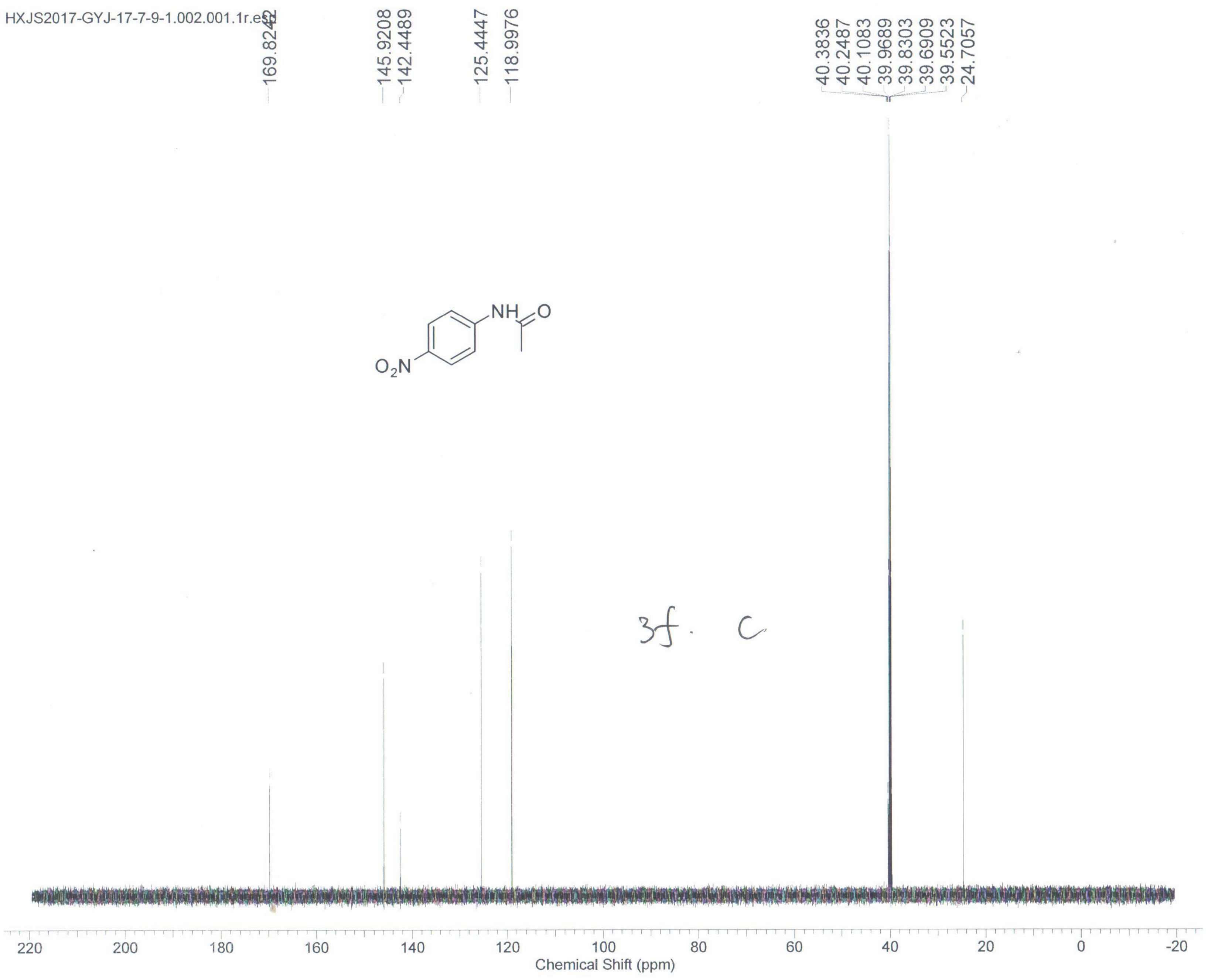

Acquisition Time (sec)	1.8175
Comment	13C NMR
Date	13 Jul 2017 11:18:24
Date Stamp	13 Jul 2017 11:18:24
Frequency (MHz)	150.90
Nucleus	13C
Number of Transients	100
Origin	spect
Original Points Count	65536
Owner	root
Points Count	262144
Pulse Sequence	zgpg30
Receiver Gain	197.01
SW(cyclical) (Hz)	36057.69
Solvent	DMSO-d6
Spectrum Offset (Hz)	15089.0967
Spectrum Type	STANDARD
Sweep Width (Hz)	36057.55
Temperature (degree C)	22.885

3604.001.001.1r.esp

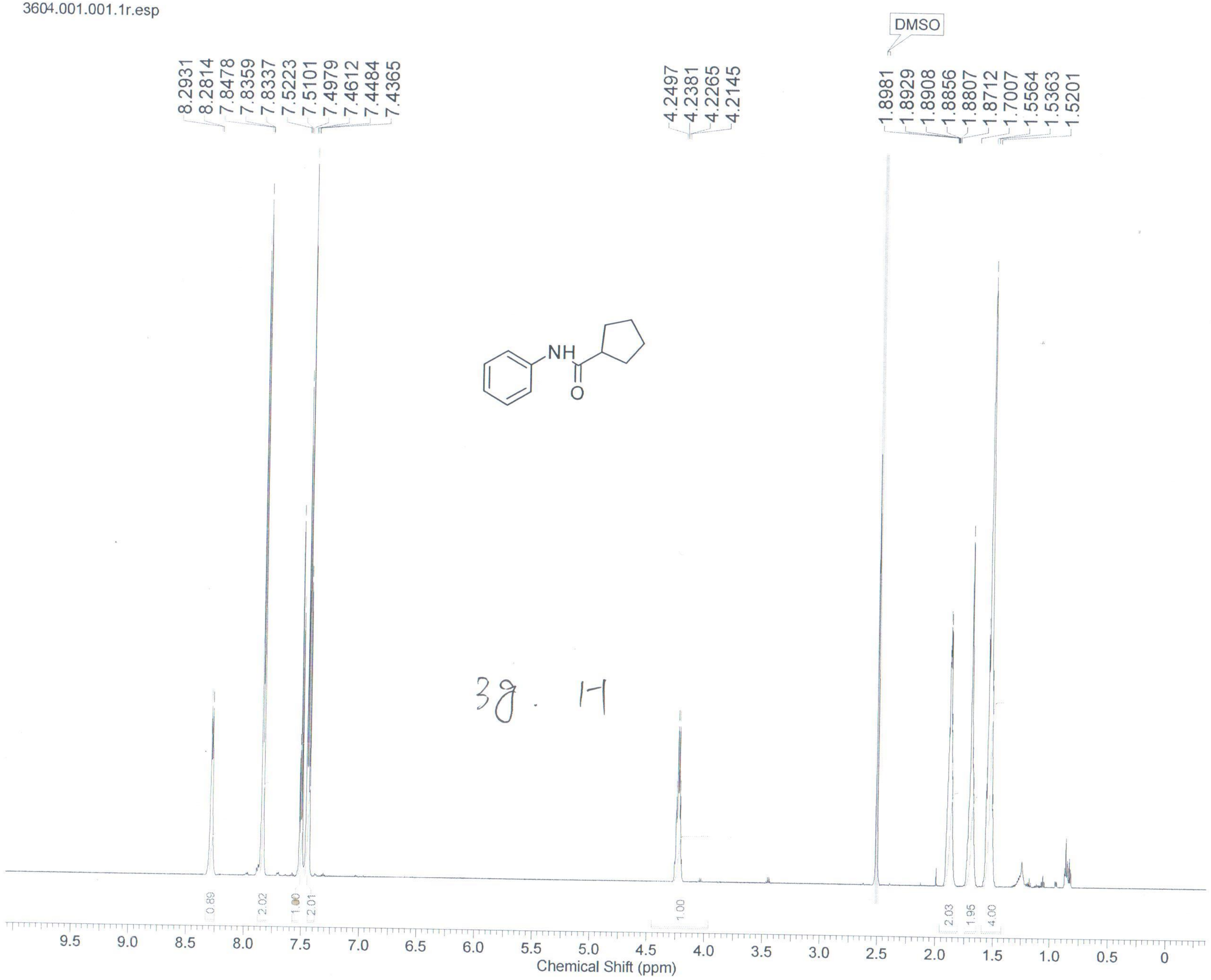

Acquisition Time (sec)	2.7263
Comment	1H NMR
Date	15 Jul 2017 07:49:20
Date Stamp	15 Jul 2017 07:49:20
Frequency (MHz)	600.13
Nucleus	1H
Number of Transients	4
Origin	spect
Original Points Count	32768
Owner	root
Points Count	65536
Pulse Sequence	zg30
Receiver Gain	49.95
SW(cyclical) (Hz)	12019.23
Solvent	DMSO-d6
Spectrum Offset (Hz)	3706.0515
Spectrum Type	STANDARD
Sweep Width (Hz)	12019.05
Temperature (degree C)	21.597

3605.002.00[illegible]1r.esp

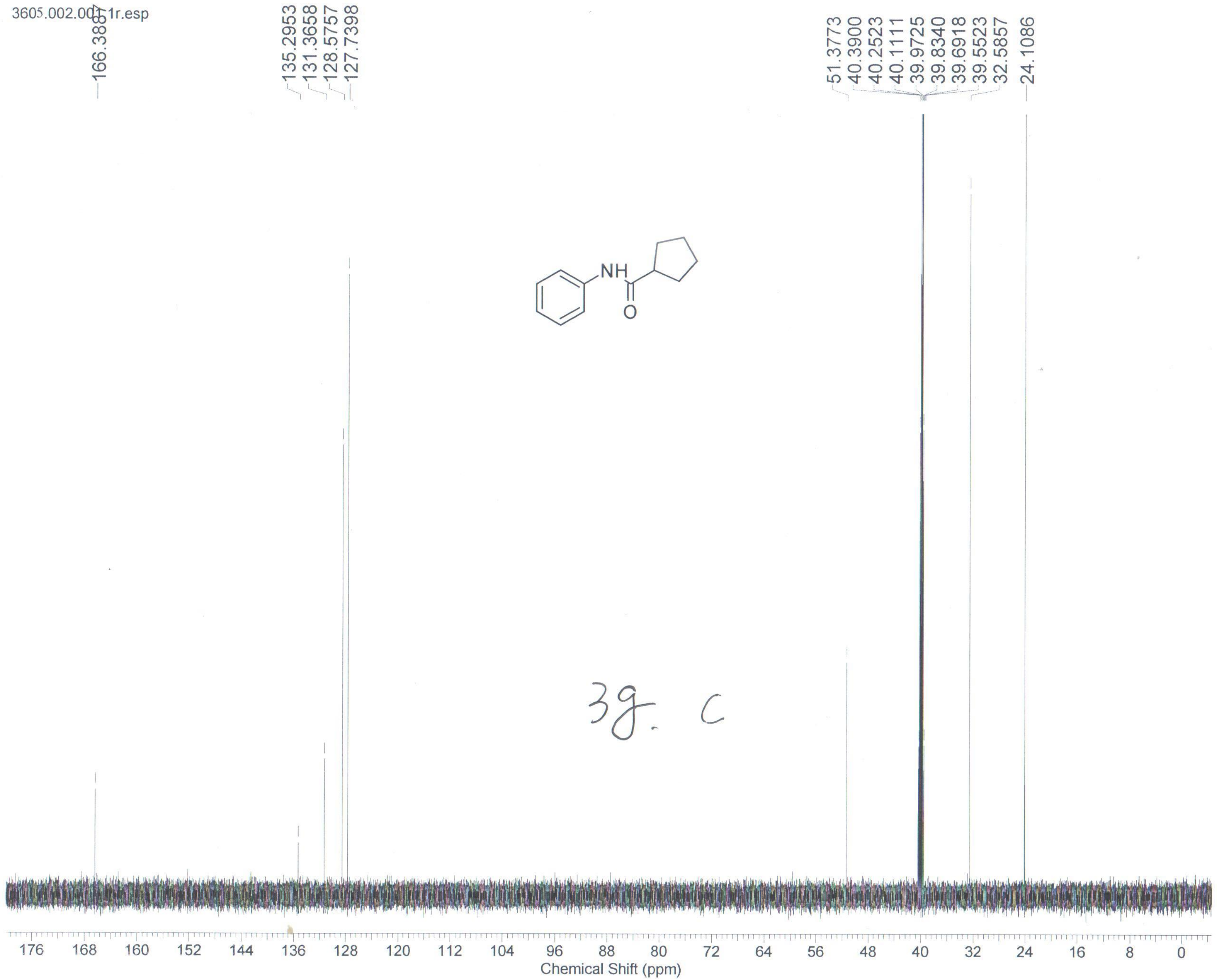

3g. C

Acquisition Time (sec)	1.8175
Comment	13C NMR
Date	15 Jul 2017 07:55:44
Date Stamp	15 Jul 2017 07:55:44
Frequency (MHz)	150.90
Nucleus	13C
Number of Transients	73
Origin	spect
Original Points Count	65536
Owner	root
Points Count	262144
Pulse Sequence	zgpg30
Receiver Gain	197.01
SW(cyclical) (Hz)	36057.69
Solvent	DMSO-d6
Spectrum Offset (Hz)	15089.0967
Spectrum Type	STANDARD
Sweep Width (Hz)	36057.55
Temperature (degree C)	23.132

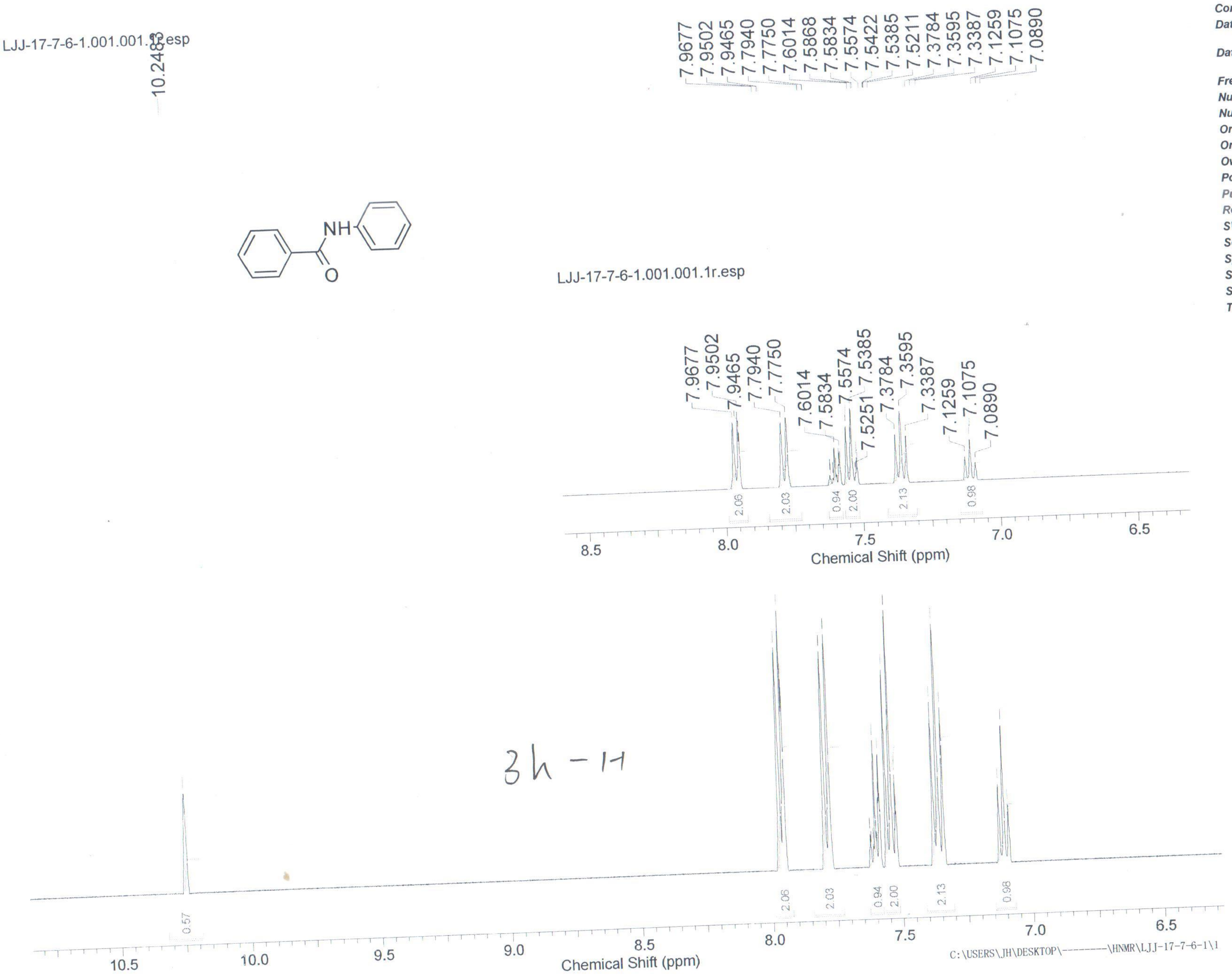

**Acquisition Time (sec)**	3.9584
**Comment**	1H
**Date**	07 Jul 2017 11:37:36
**Date Stamp**	07 Jul 2017 11:37:36
**Frequency (MHz)**	400.13
**Nucleus**	1H
**Number of Transients**	8
**Origin**	spect
**Original Points Count**	32768
**Owner**	Administrator
**Points Count**	262144
**Pulse Sequence**	zg30
**Receiver Gain**	512.00
**SW(cyclical) (Hz)**	8278.15
**Solvent**	DMSO-d6
**Spectrum Offset (Hz)**	2470.9668
**Spectrum Type**	STANDARD
**Sweep Width (Hz)**	8278.11
**Temperature (degree C)**	24.000

HXJS2017-HY-6Y.002.001.1r.esp

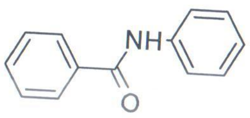

Acquisition Time (sec)	0.9088
Comment	13C NMR
Date	07 Jul 2017 14:17:36
Date Stamp	07 Jul 2017 14:17:36
Frequency (MHz)	150.90
Nucleus	13C
Number of Transients	213
Origin	spect
Original Points Count	32768
Owner	root
Points Count	32768
Pulse Sequence	zgpg30
Receiver Gain	197.01
SW(cyclical) (Hz)	36057.69
Solvent	DMSO-d6
Spectrum Offset (Hz)	15089.0967
Spectrum Type	STANDARD
Sweep Width (Hz)	36056.59
Temperature (degree C)	23.427

HXJS2017-HY-6Y.002.001.1r.esp

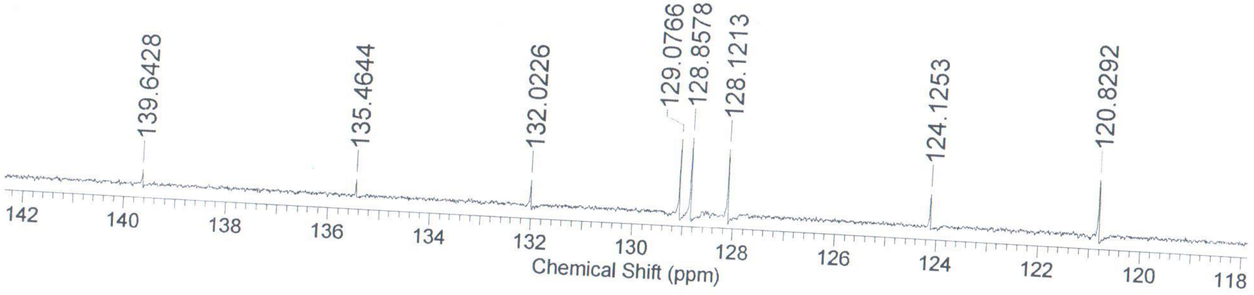

3h-c

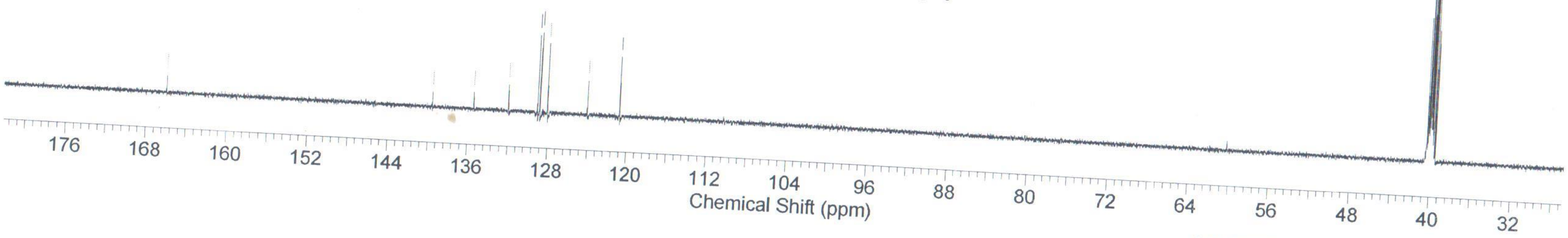

C:\USERS\JH\DESKTOP\--------\HNMR\HXJS2017-HY-6Y\2

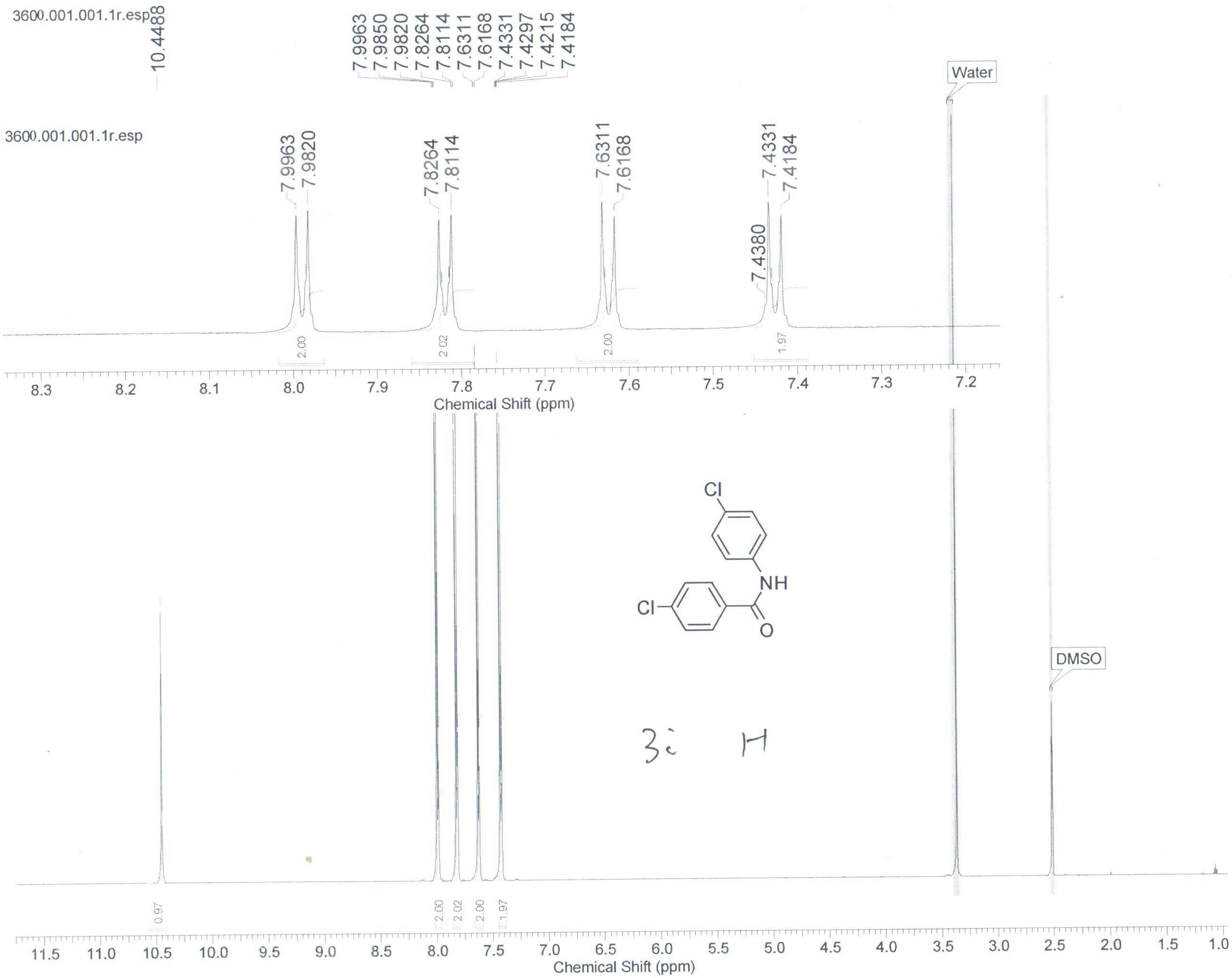

**Acquisition Time (sec)**	2.7263
**Comment**	1H NMR
**Date**	15 Jul 2017 08:02:08
**Date Stamp**	15 Jul 2017 08:02:08
**Frequency (MHz)**	600.13
**Nucleus**	1H
**Number of Transients**	4
**Origin**	spect
**Original Points Count**	32768
**Owner**	root
**Points Count**	65536
**Pulse Sequence**	zg30
**Receiver Gain**	78.68
**SW(cyclical) (Hz)**	12019.23
**Solvent**	DMSO-d6
**Spectrum Offset (Hz)**	3706.0515
**Spectrum Type**	STANDARD
**Sweep Width (Hz)**	12019.05
**Temperature (degree C)**	21.737

3601.002.001.1r.esp

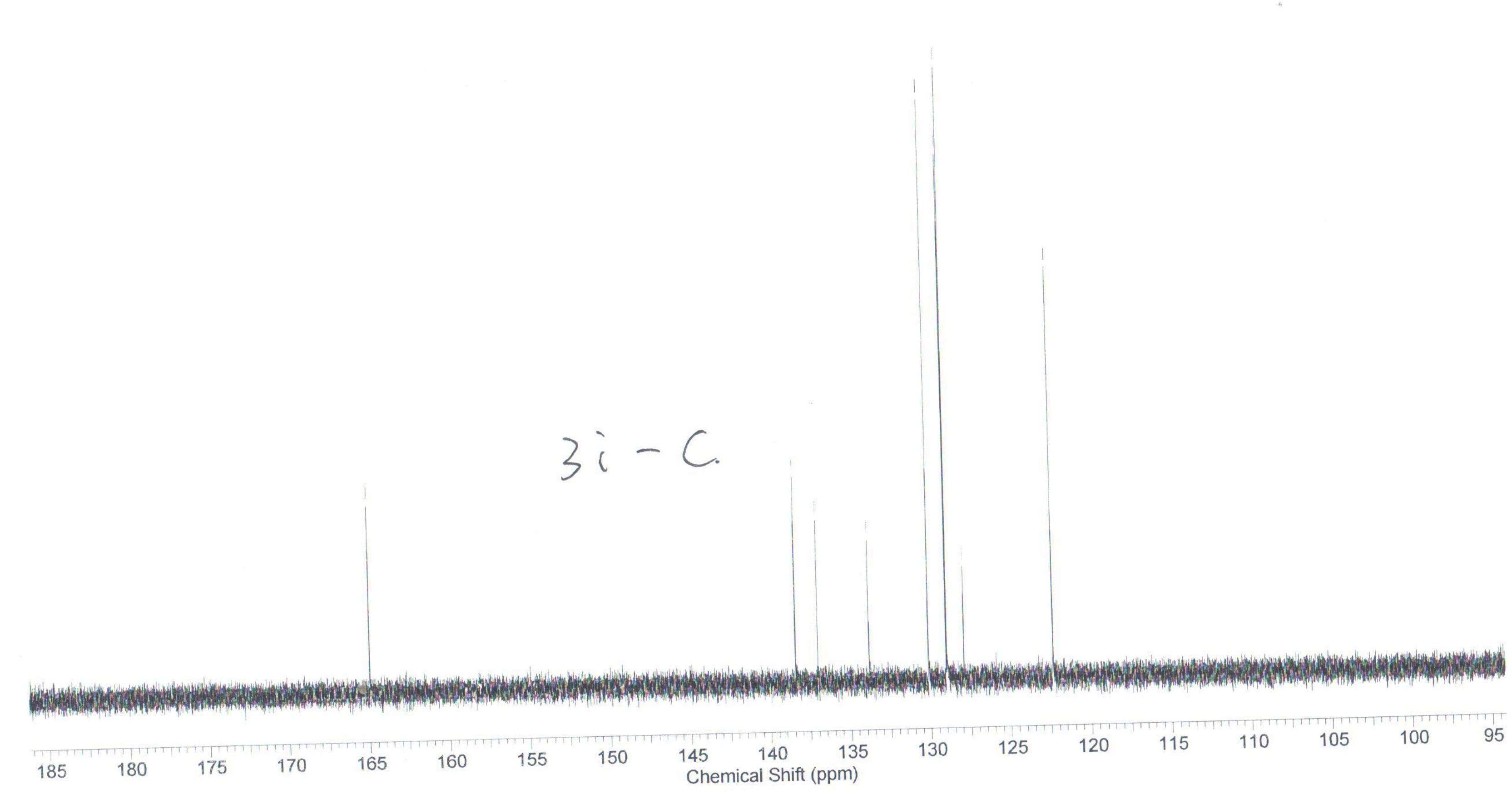

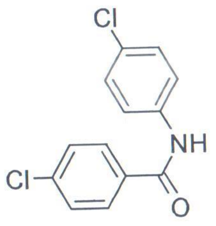

**Acquisition Time (sec)**	1.8175
**Comment**	13C NMR
**Date**	15 Jul 2017 08:04:16
**Date Stamp**	15 Jul 2017 08:04:16
**Frequency (MHz)**	150.90
**Nucleus**	13C
**Number of Transients**	77
**Origin**	spect
**Original Points Count**	65536
**Owner**	root
**Points Count**	262144
**Pulse Sequence**	zgpg30
**Receiver Gain**	197.01
**SW(cyclical) (Hz)**	36057.69
**Solvent**	DMSO-d6
**Spectrum Offset (Hz)**	15089.0967
**Spectrum Type**	STANDARD
**Sweep Width (Hz)**	36057.55
**Temperature (degree C)**	22.585

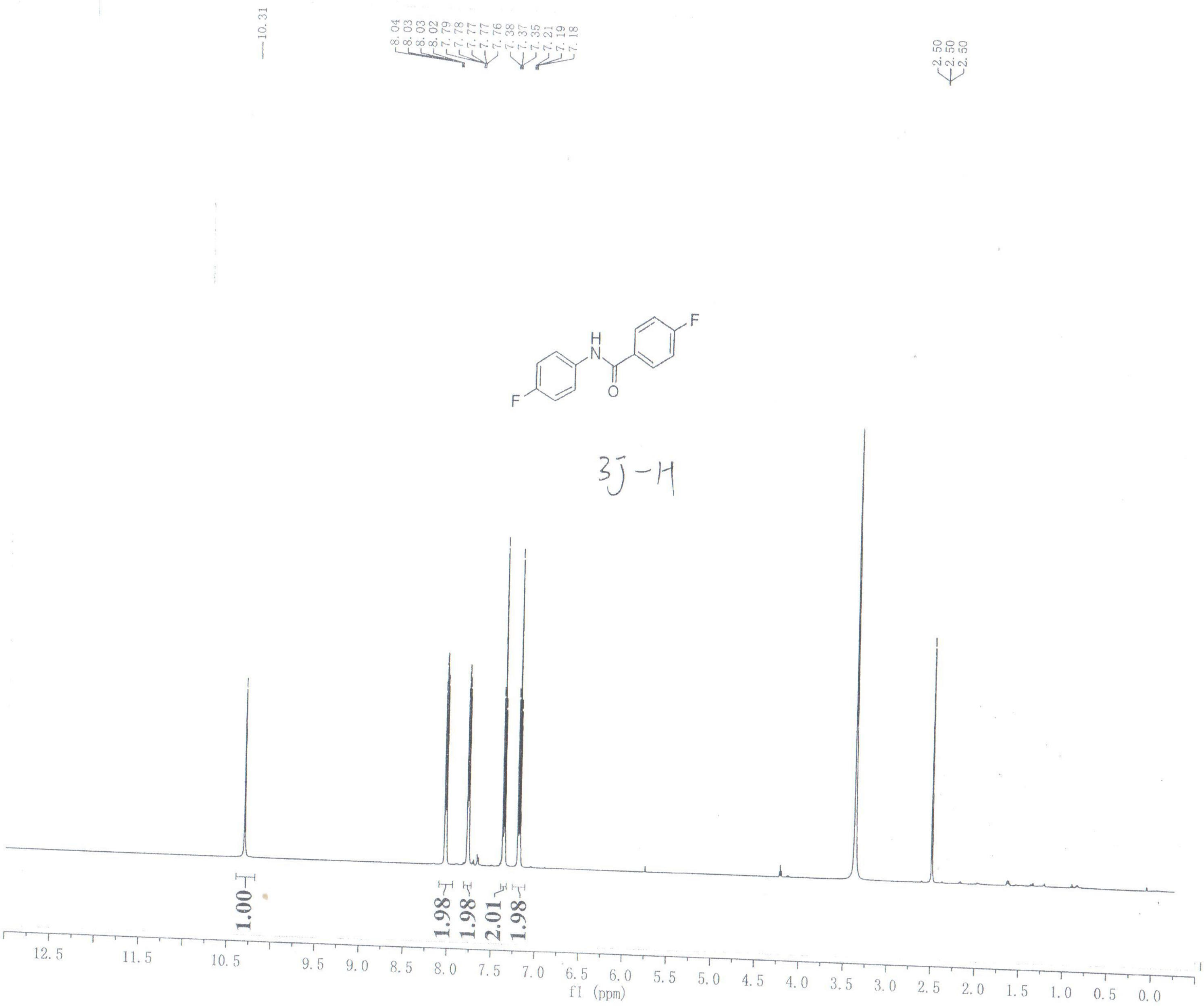
10.31
8.04 8.03 8.03 8.02 7.79 7.78 7.77 7.77 7.76 7.38 7.37 7.35 7.21 7.19 7.18
2.50 2.50 2.50
F
H
N
O
F
3j-H
1.00
1.98
1.98
2.01
1.98
12.5 11.5 10.5 9.5 9.0 8.5 8.0 7.5 7.0 6.5 6.0 5.5 5.0 4.5 4.0 3.5 3.0 2.5 2.0 1.5 1.0 0.5 0.0
f1 (ppm)

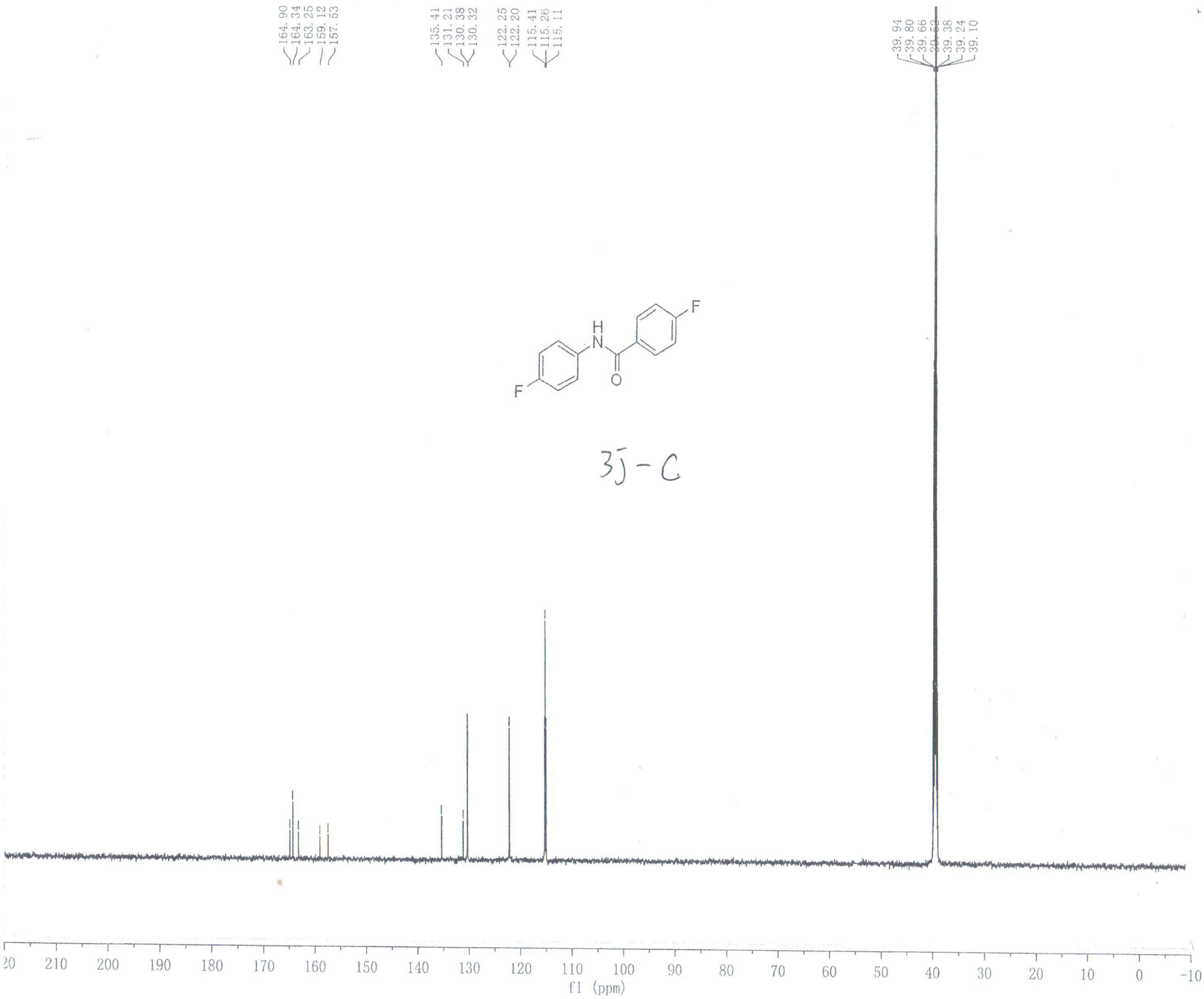
164.90
164.34
163.25
159.12
157.53
135.41
131.21
130.38
130.32
122.25
122.20
115.41
115.26
115.11
39.94
39.80
39.66
39.38
39.24
39.10
F
H
N
O
F
3j-C
f1 (ppm)

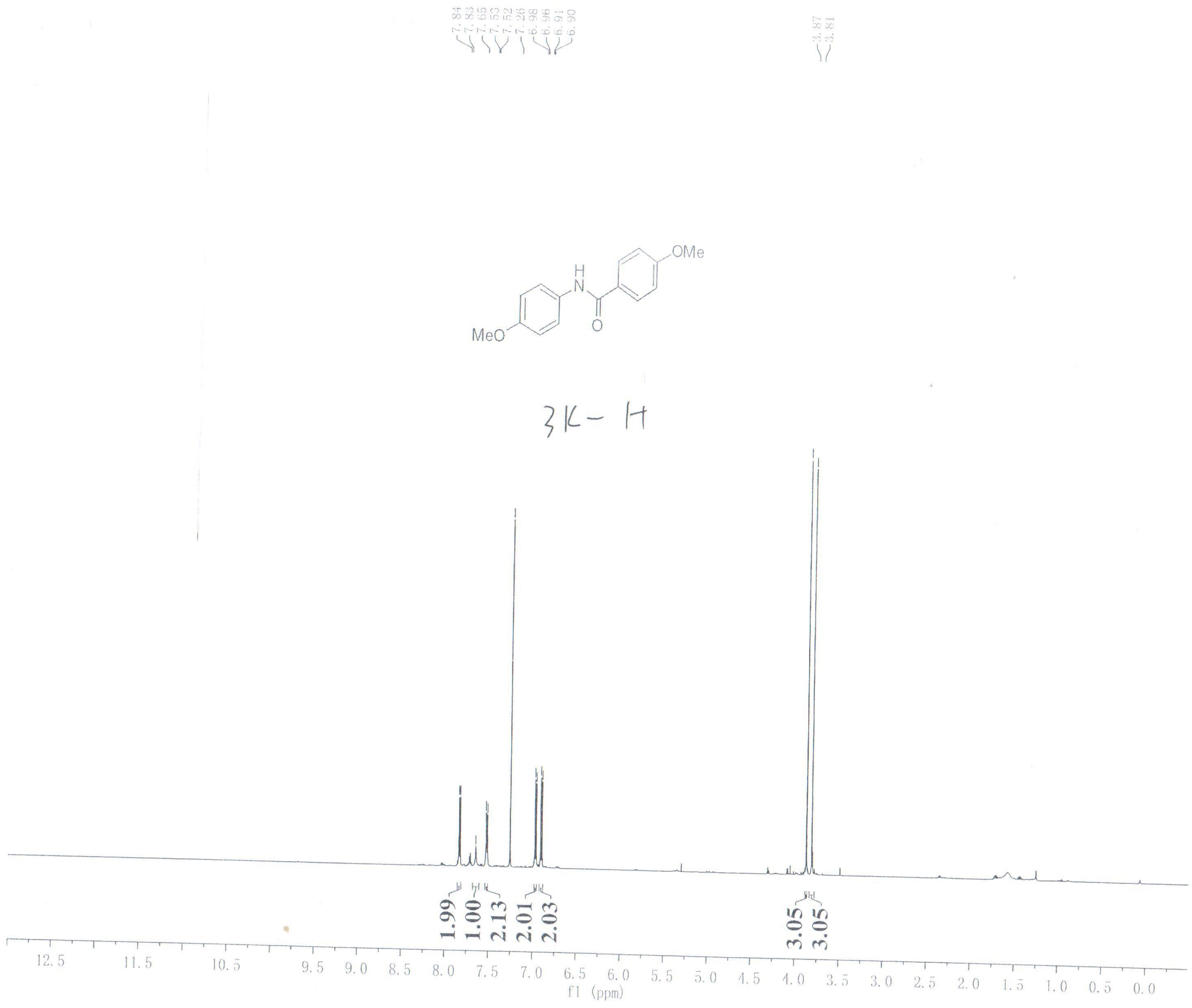
7.84
7.83
7.65
7.53
7.52
7.26
6.98
6.96
6.91
6.90
3.87
3.81
OMe
H
N
O
MeO
3K- H
1.99
1.00
2.13
2.01
2.03
3.05
3.05
12.5
11.5
10.5
9.5
9.0
8.5
8.0
7.5
7.0
6.5
6.0
5.5
5.0
4.5
4.0
3.5
3.0
2.5
2.0
1.5
1.0
0.5
0.0
f1 (ppm)

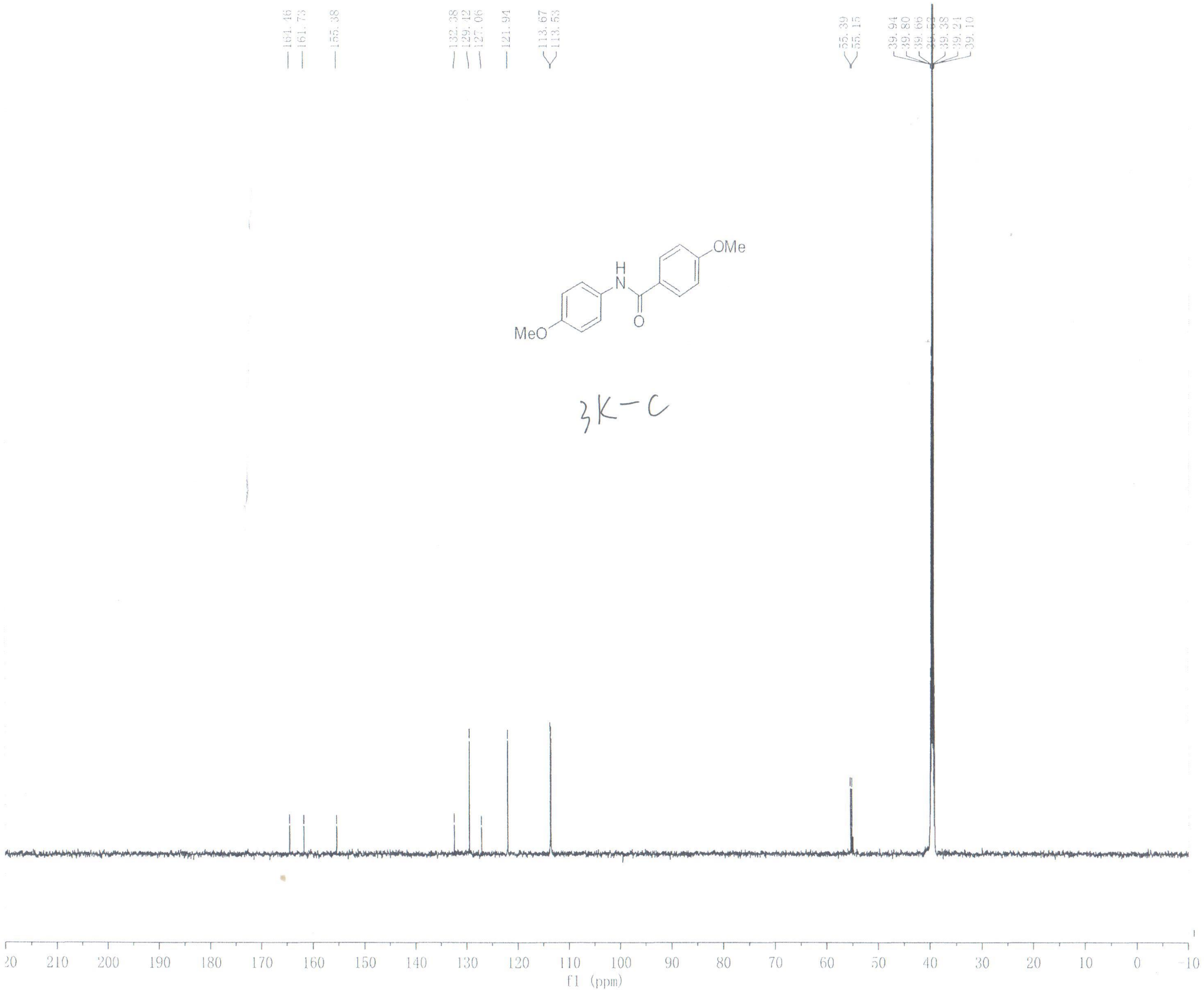

164.46
161.73
155.38
132.38
129.42
127.06
121.94
113.67
113.53
55.39
55.15
39.94
39.80
39.66
39.38
39.21
39.10
MeO
H
N
O
OMe
3K-C
f1 (ppm)

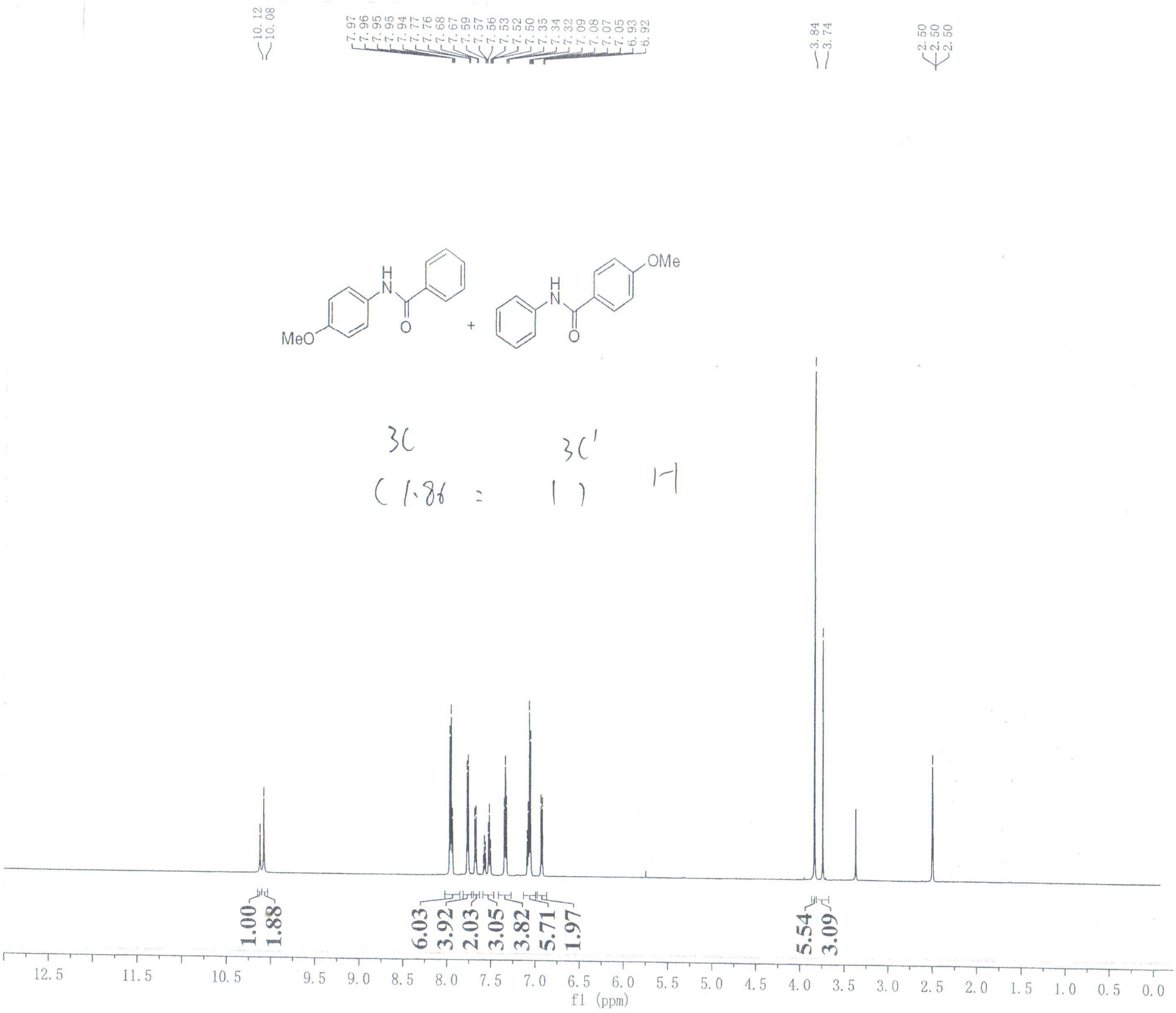
10.12
10.08
7.97
7.96
7.95
7.95
7.94
7.77
7.76
7.68
7.67
7.59
7.57
7.56
7.53
7.52
7.50
7.35
7.34
7.32
7.09
7.08
7.07
7.05
6.93
6.92
3.84
3.74
2.50
2.50
2.50
MeO
H
N
O
+
OMe
3c
3c'
(1.86 = 1)
1.00
1.88
6.03
3.92
2.03
3.05
3.82
5.71
1.97
5.54
3.09
12.5
11.5
10.5
9.5
9.0
8.5
8.0
7.5
7.0
6.5
6.0
5.5
5.0
4.5
4.0
3.5
3.0
2.5
2.0
1.5
1.0
0.5
0.0
f1 (ppm)

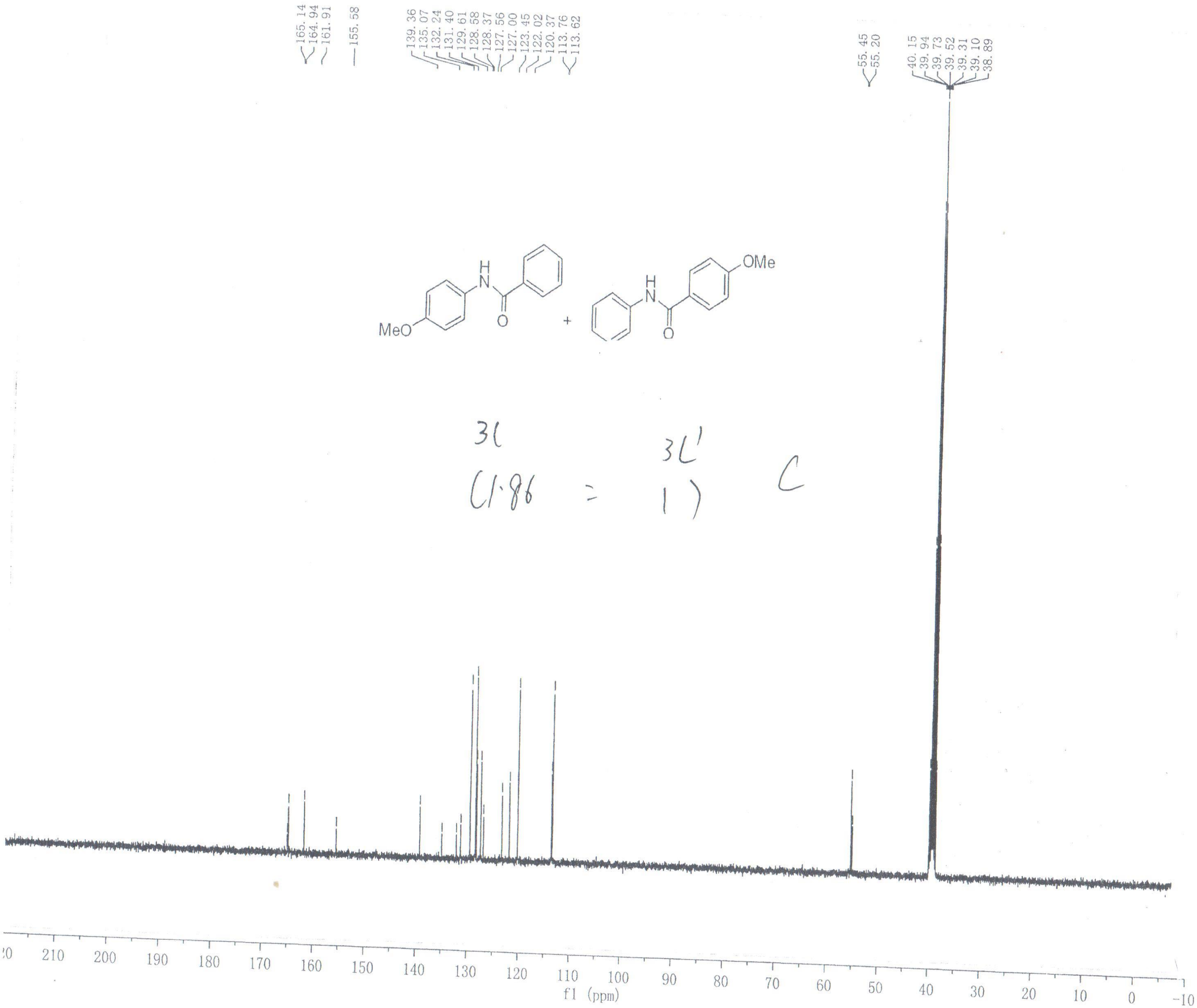
165.14
164.94
161.91
155.58
139.36
135.07
132.24
131.40
129.61
128.58
128.37
127.56
127.00
123.45
122.02
120.37
113.76
113.62
55.45
55.20
40.15
39.94
39.73
39.52
39.31
39.10
38.89
MeO
OMe
3c
3c'
(1.86 = 1)
C
f1 (ppm)

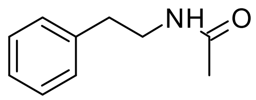

3m-H

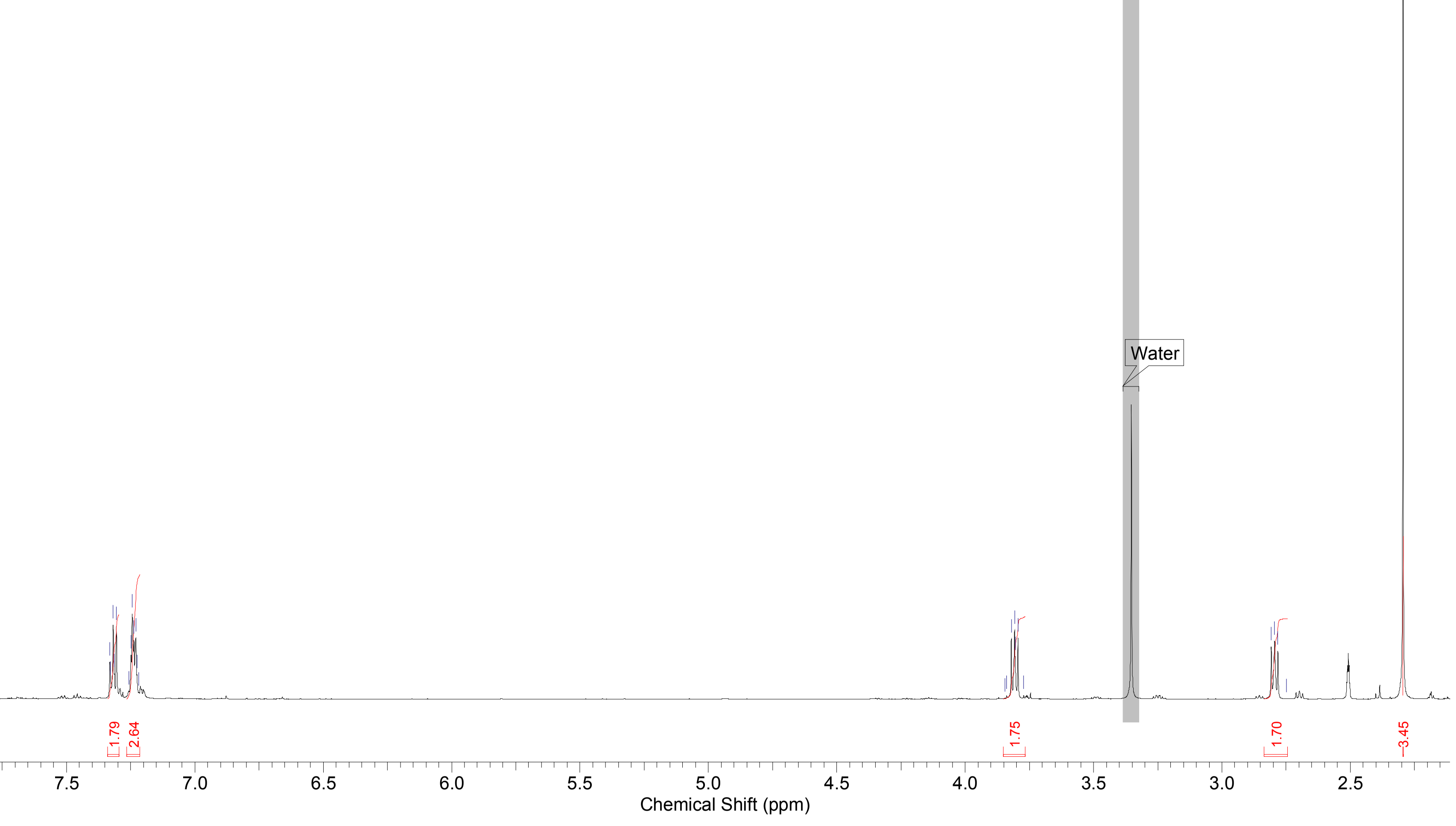
3.8457
3.8390
3.8203
3.8075
3.8053
3.7943
3.7720
2.8079
2.7947
2.7822
2.7492
2.2948
Water
1.79
2.64
1.75
1.70
3.45
7.5
7.0
6.5
6.0
5.5
5.0
4.5
4.0
3.5
3.0
2.5
Chemical Shift (ppm)

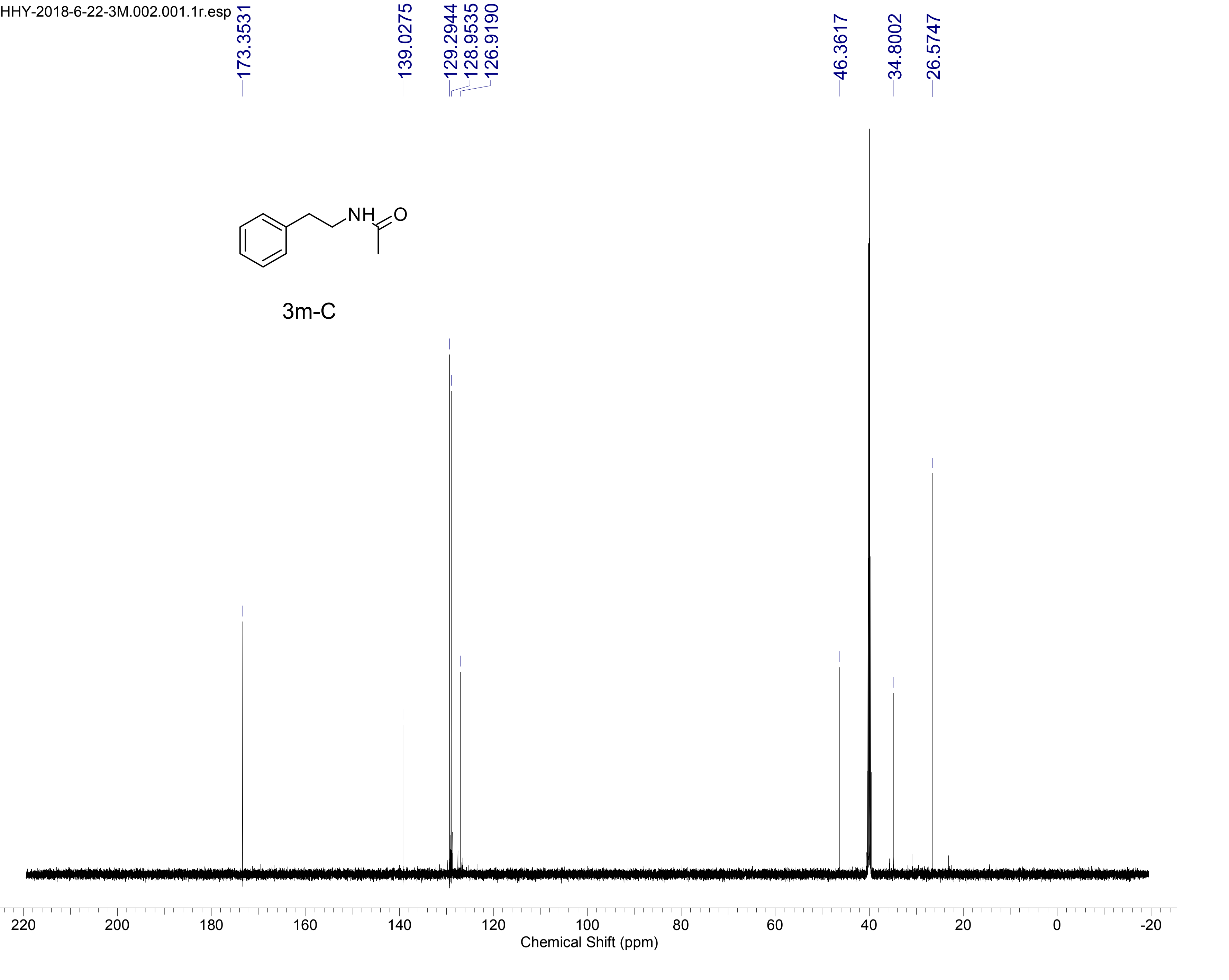

HHY-2018-6-22-3M.002.001.1r.esp
173.3531
139.0275
129.2944
128.9535
126.9190
46.3617
34.8002
26.5747
NH
O
3m-C
220
200
180
160
140
120
100
80
60
40
20
0
-20
Chemical Shift (ppm)

4O.001.001.1r.esp

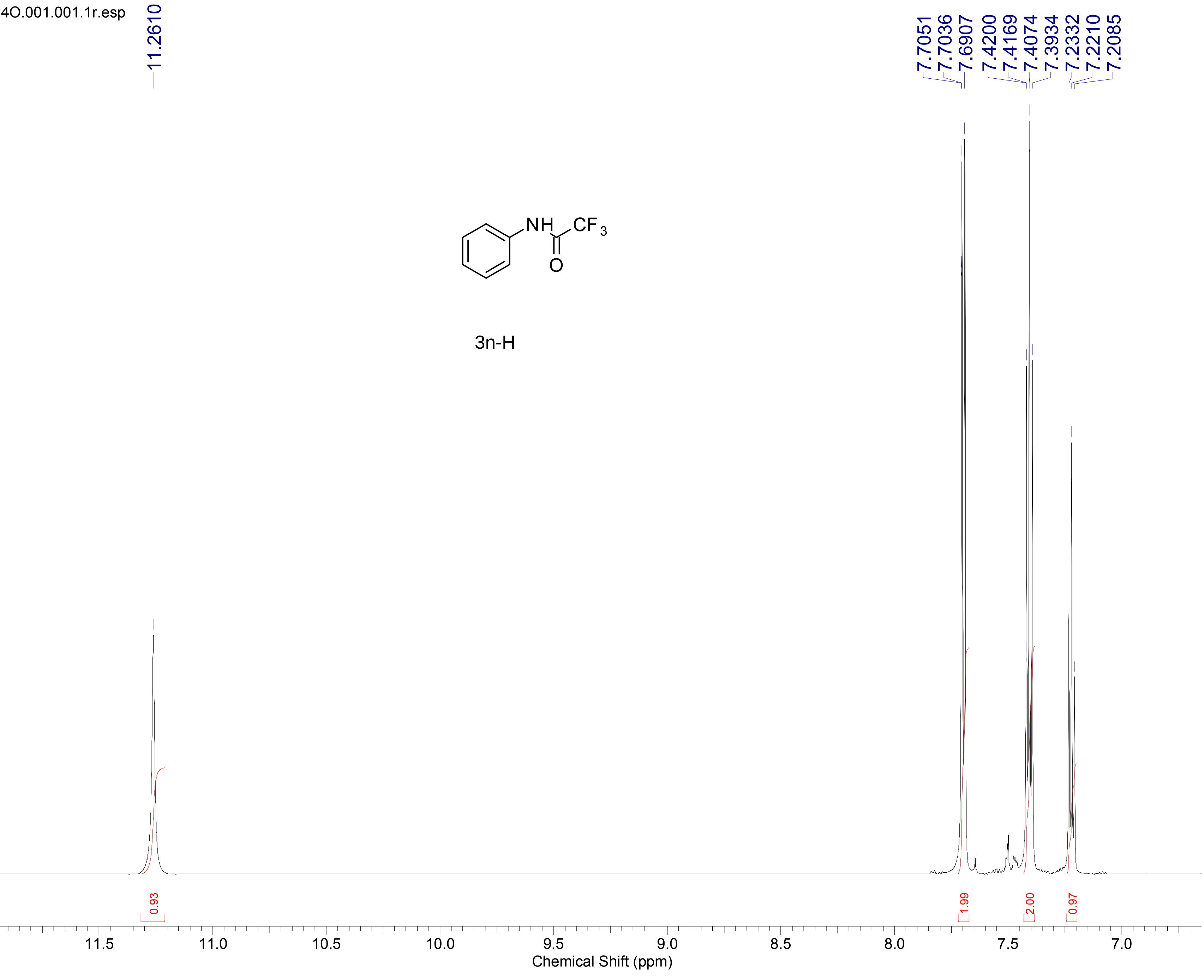

4O.002.001.1r.esp

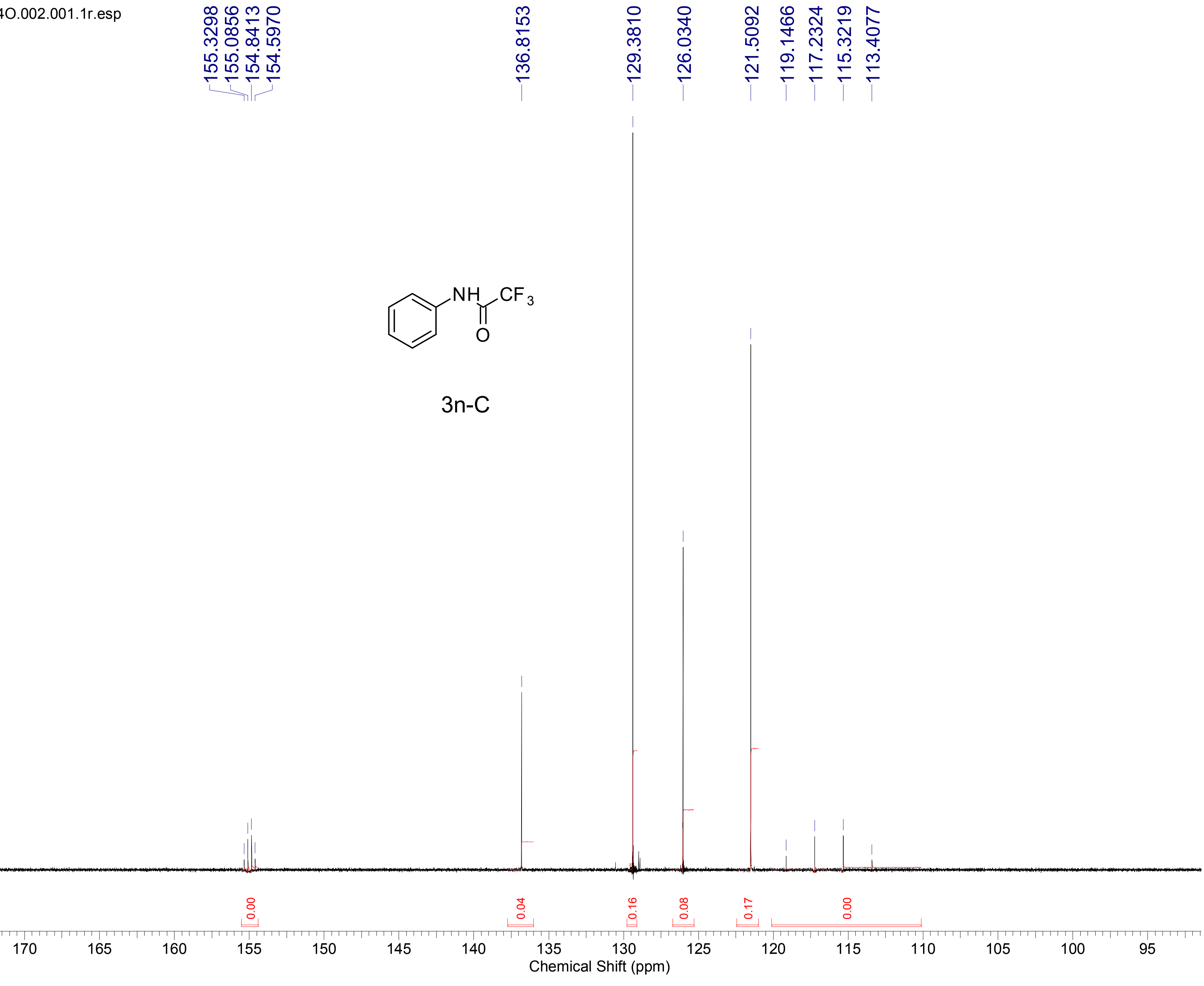

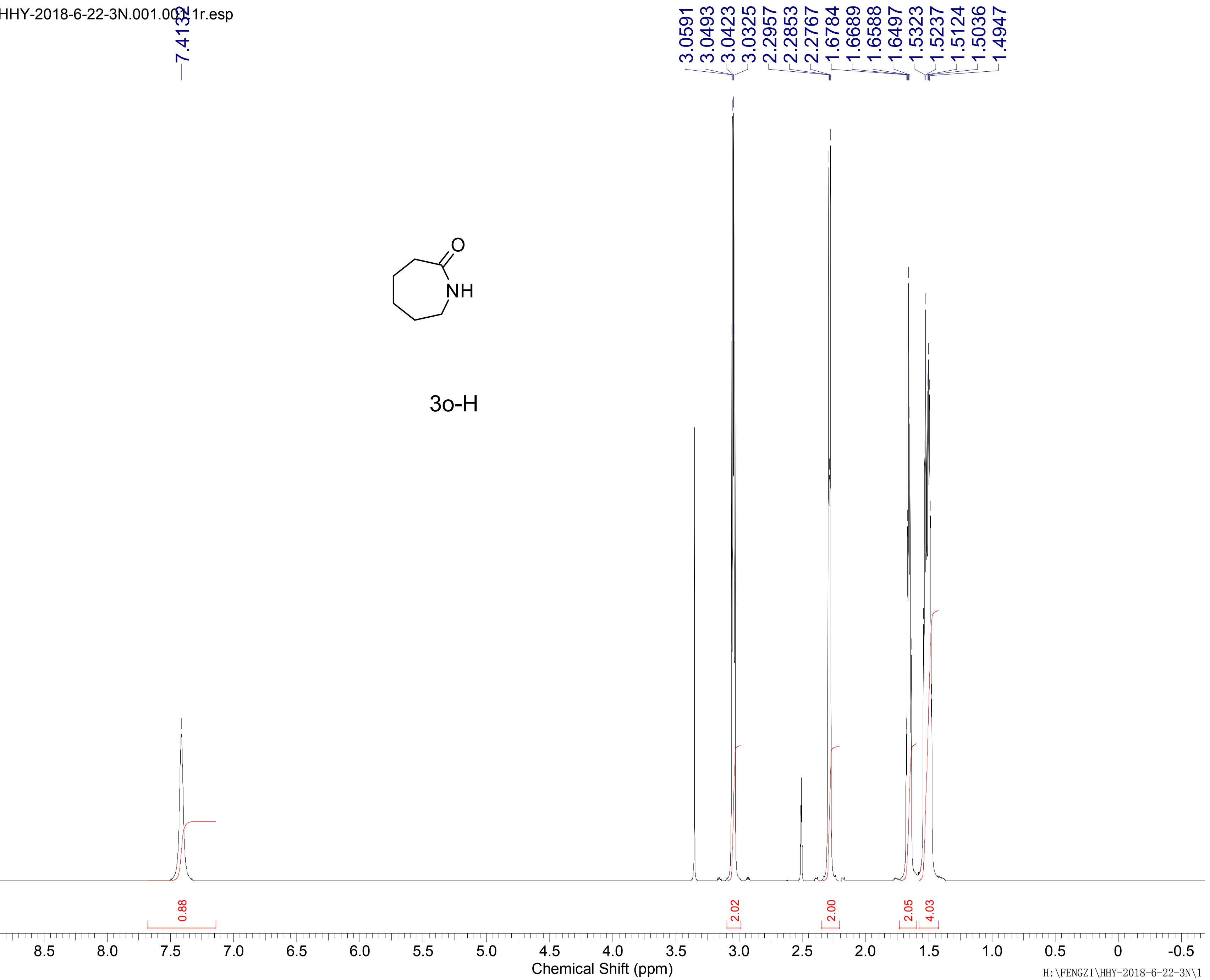
HHY-2018-6-22-3N.001.001.1r.esp
7.4132
3.0591
3.0493
3.0423
3.0325
2.2957
2.2853
2.2767
1.6784
1.6689
1.6588
1.6497
1.5323
1.5237
1.5124
1.5036
1.4947
O
NH
3o-H
0.88
2.02
2.00
2.05
4.03
8.5 8.0 7.5 7.0 6.5 6.0 5.5 5.0 4.5 4.0 3.5 3.0 2.5 2.0 1.5 1.0 0.5 0 -0.5
Chemical Shift (ppm)
H:\FENGZI\HHY-2018-6-22-3N\1

HHY-2018-6-22-3N.002.001.1r.esp

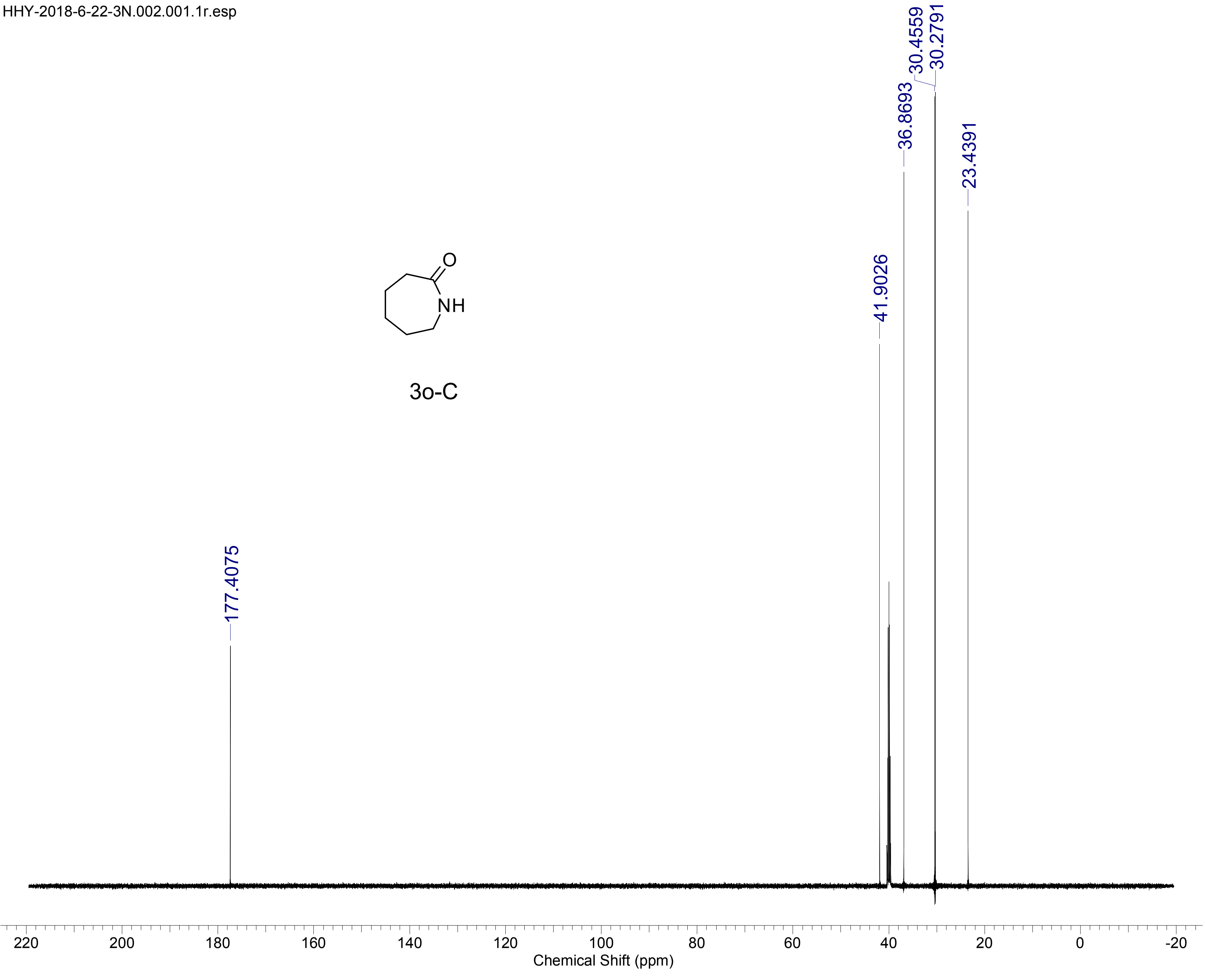

3o-C
